# The Early Years of 2,2′-Bipyridine—A Ligand in Its Own Lifetime

**DOI:** 10.3390/molecules24213951

**Published:** 2019-10-31

**Authors:** Edwin C. Constable, Catherine E. Housecroft

**Affiliations:** Department of Chemistry, University of Basel, BPR 1096, Mattenstrasse 24a, CH-4058 Basel, Switzerland; catherine.housecroft@unibas.ch

**Keywords:** 2,2′-bipyridine, synthesis, coordination chemistry, supramolecular chemistry, history

## Abstract

The first fifty years of the chemistry of 2,2′-bipyridine are reviewed from its first discovery in 1888 to the outbreak of the second global conflict in 1939. The coordination chemistry and analytical applications are described and placed in the context of the increasingly sophisticated methods of characterization which became available to the chemist in this time period. Many of the “simple” complexes of 2,2′-bipyridine reported in the early literature have been subsequently shown to have more complex structures.

## 1. Introduction

**Figure 1:**
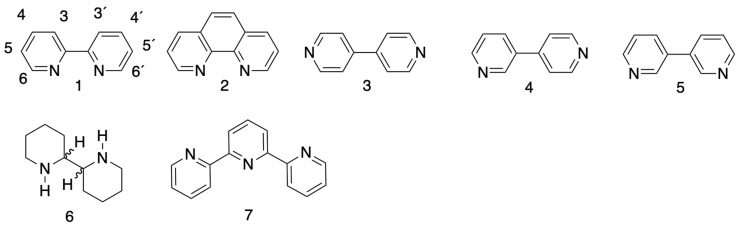
The structures of 2,2′-bipyridine (**1**) and some related compounds. The numbering scheme for the parent compound, 2,2′-bipyridine is shown. The coordination chemistry of 1,10-phenanthroline (**2**) is very similar to that of **1**. The compounds **3**, **4** and **5** are the isomers 4,4′-bipyridine, 3,4-bipyridine and 3,3’-bipyridine. Compound **6**, 2,2′-bipiperidine, is one of the reduction products of **1**. Also shown is the next higher oligopyridine 2,2′:6’,2”-terpyridine, **7**, which Blau described in 1889.

2,2′-Bipyridine (bpy, **1**, Figure 1) celebrated its 131st birthday in 2019 and is one of the most commonly used and most easily identified ligands in coordination chemistry. Coordination compounds incorporating **1** have played crucial roles in developing our understanding of the thermodynamics and kinetics of complexation of metal ions, the bonding, photochemistry, photophysics and electrochemistry of metal complexes. As a bidentate metal-binding domain, bpy has also found widespread application as a scaffold in supramolecular and metallosupramolecular chemistry. Indeed, at the turn of the millennium, 2,2′-bipyridine was described as “the most widely used ligand” [1] a status that has changed little in the intervening two decades. Not surprisingly, this commonly used type of ligand, together with the higher oligopyridines and the closely related species 1,10-phenanthroline, **2** (Figure 1), has been the subject of a number of reviews [2,3,4,5,6,7,8] since the first review of their coordination chemistry in 1954 [9]. 2,2′-Bipyridine is the first member of a series of higher oligopyridines, which can also act as polydentate ligands for metal-centres [10,11,12,13,14,15]. This review aims to present a comprehensive overview of the first 50 years of the chemistry of **1** and its simple derivatives up to 1939; the patent literature is not included in the survey and the chemistry of the higher oligopyridines and the 1,10-phenanthrolines is not covered in detail. The material is presented in a chronological manner with the intention of emphasizing how developments and trends in chemical science paralleled technical advances and societal needs and demands. An additional emphasis is on the coordination chemistry and the observations that many “simple” compounds reported in this early period were subsequently shown to have more complex structures. No explicit reference is cited for compounds where structural data have been lodged with the Cambridge Structural Database but not otherwise published.

## 2. Discovery and the Discoverer

**Figure 2:**
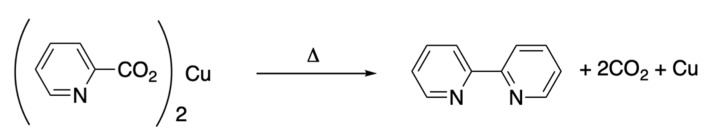
Fritz Blau first prepared 2,2′-bipyridine by the dry distillation of copper(II) pyridine-2-carboxylate [16].

Fritz Blau described the dry distillation of copper(II) pyridine-2-carboxylate in 1888 and reported the loss of gases including hydrogen cyanide and the formation of a distillate containing pyridine and a new base with melting point 70 °C (Figure 2). This latter compound gave an intense red colour with FeSO_4_ (eine intensiv rothe Färbung mit Eisenvitriol) [16]. He assigned the correct structure of **1** to the new compound which he described as *α-α-Dipyridyl* and noted that one might obtain isomeric *Dipyridyle* by distilling either mixtures of pyridine-2-carboxylate, pyridine-3-carboxylate and pyridine-4-carboxylate salts or mixed salts containing these anions. One comment that Blau makes is not so easy to understand “The substance with a melting point of 70° causes the iron reaction to become weaker the purer it is, without the colouring being completely suppressed (Die Substanz vom Schmelzpunkt 70° giebt die Eisenreaction umso schwächer, je reiner sie ist, ohne dass die Färbung ganz unterdrückt werden könnte). The yield of 2,2′-bipyridine is typically less than 20%.

It seems likely that Weidel had obtained 2,2′-bipyridine earlier by the distillation of a mixture of calcium pyridine-2-carboxylate and calcium oxide [17], although he identifies his product with the “dipyridine” isolated by Anderson from the reaction of pyridine with sodium [18,19]. The initial product of the reaction of sodium with pyridine is a blue “sodium dipyridine”. The major isolated product of this reaction, after oxidative and hydrolytic workup, was later shown by Weidel to be 4,4′-bipyridine *(γ-Dipyridyl*), 3 [20]. However, the reaction of sodium with pyridine is complex and the major isolated products are typically **1** and **2**, with the 4,4′-isomer, **3** (Figure 1) dominant [21]. Modified conditions for the reaction of sodium with pyridine followed by subsequent oxidation with dry or moist air gave not only **1** and **2**, but also 3,4-bipyridine (**4**, Figure 1); heating the reaction mixture before oxidation gave 3,3′-bipyridine (**5**, Figure 1) amongst the products [22]. In an 1885 publication, Hartley reports the absorption spectra of a series of aromatic and heteroaromatic compounds, including an unidentified “dipyridine” that he had obtained from a Dr Ramsay in Bristol [23], but this was probably Anderson’s “dipyridine”. Heuser and Stoehr extended the studies of the reaction with sodium to 2-methylpyridine and report the isolation of a compound which they described as *αα-Dimethyldipyridyl* and which can be confidently identified as 2,2′-dimethyl-4,4′-bipyridine [24,25,26].

Blau published one additional paper describing the dry distillation of copper(II) pyridine-2-carboxylate [27]. In these publications, he confirmed the formation of an intense red colouration when bpy reacted with iron(II) salts, described the formation of an insoluble hexacyanoferrate(IV) salt of the free ligand, and reported various reactions of bpy. Quarternization gave *N,N*′-dimethyl-2,2′-bipyridinium iodide as a yellow solid which did not give the typical colour reaction with iron(II). Reduction with sodium and 3-methylbutanol gave 2,2′-bipiperidine (**6**, Figure 1). One aspect of Blau’s work that seems to have been unnoticed is his description of a less volatile product of the distillation of copper(II) pyridine-2-carboxylate. This compound gives an intense purple colouration with iron(II) salts and has a very similar elemental constitution to bpy. Blau proposed that it had “more than two pyridine rings” [27]. Although Blau mentions *Tetrapyridyls*, this compound can actually be nothing other than 2,2′:6’,2”-terpyridine (**7**, Figure 1), the first preparation of which is normally credited to Morgan and Burstall. Blau also used bpy as one of the test substances for his detailed publication on methods for combustion analysis, also published in 1889 [28].

Some ten years later, Blau published a new paper *Über neue organische Metallverbindungen* in which he describes many of the features that define the coordination chemistry of bpy [29]. Blau established the 3:1 bpy-iron stoichiometry for the red compound (which contains the cation we now formulate as [Fe(bpy)_3_]^2+^) and, building on the paradigm-shifting 1893 vision of Werner [30], formulated the compounds *Tridipyridylferrosalze*). He describes the oxidation to the blue *Tridipyridyl-ψ-Ferrisalze* [Fe(bpy)_3_]^3+^ (*ψ*, meaning pseudo, *“*because they are not obtainable by the combination of dipyridyl and iron oxide salts”) together with the preparation of [Ni(bpy)_3_]^2+^ and [Co(bpy)_3_]^2+^ salts. Blau also reports that copper(II), cadmium(II) and chromium(III) complexes could be formed but noted that he could not obtain compounds with manganese, lead, or aluminium salts.

**Figure 3:**
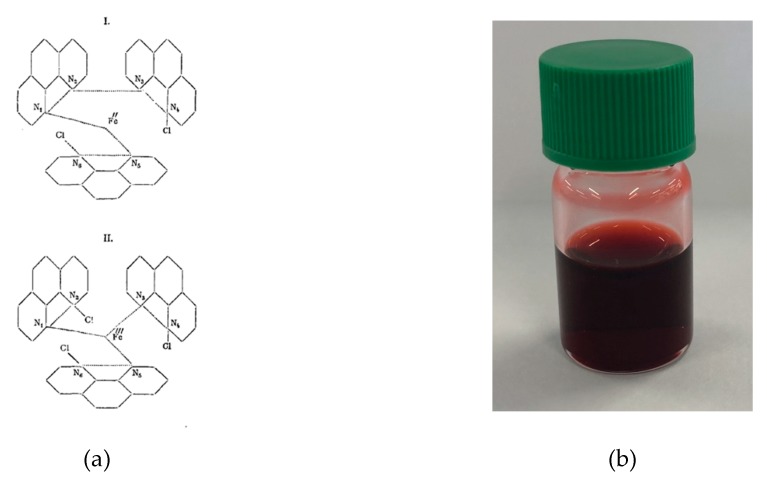
(**a**) The structures proposed by Fritz Blau for [Fe(phen)_3_]^2+^ and [Fe(phen)_3_]^3+^ [29]. The diagrams indicate that Blau had not fully understood Werner’s concepts of primary and secondary valence. The bonding to the iron(II) and iron(III) centres are based upon valency of two and three respectively (rather than an oxidation state). The bonding of the chloride to the nitrogen was relatively common in the depiction of ammonium and pyridinium salts at this period in time. (Reprinted from reference 29 by permission from Springer). (**b**) The bright red colour of the [Fe(bpy)_3_]^2+^ is familiar to all scientists who work with 2,2′-bipyridines.

Blau also clearly identifies the challenges associated with kinetic or thermodynamic stability inherent in coordination chemistry “If, on the other hand, a dipyridyl solution contains a sufficient amount of a zinc or cadmium salt, ferrosulphate [FeSO_4_] does not produce any colouring at all, whereas the red solution of a tridipyridylferro salt is not altered by the subsequent addition of one of the above salts, so that it is by no means the same whether a dipyridyl solution is first treated with iron and then zinc vitriol, or first zinc and then iron vitriol”. In the case of copper(II), he described complexes with 1:1 and 2:1 bpy:Cu composition. This paper not only introduced bpy coordination chemistry to the world, but also describes the first preparation of 1,10-phenanthroline (phen, *α-Phenantrolin*) from a double Skraup synthesis of benzene-1,4-diamine with propane-1,2,3-triol as well as the corresponding iron, cobalt and nickel [M(phen)_3_]^2+^ complexes (Figure 3a).

Blau did not publish any further significant work in coordination chemistry, but began work at Auer in Berlin in 1902. He went on to become the chief research advisor to Osram after its foundation in 1919, with responsibility for the patent department, the research department and the Research Society for Electric lighting, holding the latter position until his death in 1929 [31,32,33]. Although he published no further research papers in chemistry, he was a prolific innovator and is the originator of some 185 patents in a wide range of areas. In view of the importance of bpy and its derivatives in a vast range of photonic and photoactive materials used, inter alia, for lighting and other technological applications, I like to think that Fritz Blau would be proud of his legacy.

## 3. Fundamentals

### 3.1. Nomenclature

Compound **1** has a PIN (preferred IUPAC nomenclature) name of 2,2′-bipyridine [34] and a recommended abbreviation as a ligand of bpy [35]. Similarly, compound **2** has a PIN name of 1,10-phenanthroline [36] and a recommended abbreviation as a ligand of phen [35]. Over the years, **1** has had a variety of other names including 2,2′-dipyridyl, α,α′-dipyridyl, α,α-dipyridyl, 2,2′-dipyridine, 2,2′-dipyridene, 2,2′-dipyridine, bipyridyl, 2,2′-bipyridyl, 2,2-bipyridyl, 2,2,-bipyridyl, 2,2′-bipyridyle, bipyridine, 2,6′-bipyridine, α,α′-bipyridine, 2,2′-bipyridinyl, α,α′-bipyridyl, CI-588, 2-(pyridin-2-yl)pyridine, 2-(2-pyridyl)pyridine and 2-pyridin-2-ylpyridine; the abbreviations bipy, dpy and dipy have also been used.

### 3.2. Structure

**Figure 4:**
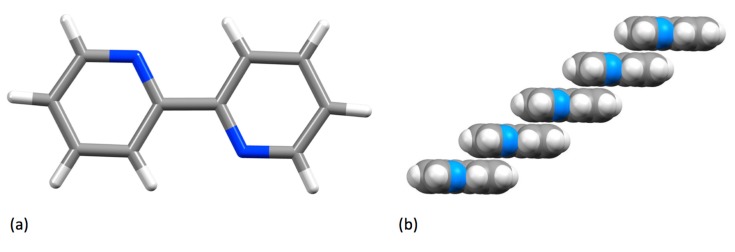
(**a**) The solid-state crystal structure of **1** shows that the molecules of 2,2′-bipyridine are planar and possess a trans-conformation; (**b**) molecules of **1** exhibit face-to-face stacking in the crystal lattice. Graphics generated from data from reference [42].

The Cambridge Structural Database (CSD) [37] contains 319 compounds containing a non-coordinated 2,2′-bipyridine molecule (CSD accessed on 8th August 2019), but in the majority of these, the molecules of **1** are involved in additional hydrogen-bonding, charge-transfer, host-guest or aromatic-aromatic interactions. Nevertheless, there are also a number of solid-state crystal structures of **1** itself [38,39,40,41,42]. In the solid state, molecules of bpy are strictly planar with a trans-conformation about the inter-annular C–C bond (Figure 4a) and exhibit both C–H...π and face-to-face (3.518 Å, Figure 4b) interactions. No polymorphs of bpy have been structurally characterized.

The monoprotonated [bpyH]^+^ cation is usually close to a *cis*-coplanar conformation with both nitrogen atoms interacting with the hydron (285 entries in CSD, 404 individual torsion data, mean 1.482°). In contrast, the diprotonated [bpyH]^+^ cation exhibits a bimodal distribution of torsion angles in the solid state, with maxima clustered around cisoid and transoid conformations (62 entries in CSD, 73 individual torsion data). The terms cisoid and transoid are used in contrast to cis and trans when referring to the related non-planar conformations.

Theoretical investigations of the conformation and the characters of the molecular orbitals of bpy have been performed at various calculational levels and an excellent overview, together with state-of-the-art DFT calculations have been presented by Alkorta et al. [43]. The planar trans conformation is found at a global minimum with a cisoid form exhibiting a 40.7° inter-annular torsion angle lying 27 kJ mol^−1^ higher in energy. The [bpyH]^+^ cation is also calculated to be planar and with a cis-conformation. In contrast, the [bpyH_2_]^2+^ dication is non-planar with a global minimum exhibiting a *transoid* structure with a torsion angle of 130.6° and a cisoid form with a torsion angle of 64.8° lying just 6.9 kJ mol^−1^ higher in energy.

### 3.3. Protonation

As discussed above, bpy can be protonated once or twice to give the (bpyH)^+^ cation and the (bpyH_2_)^2+^ dication, respectively. There is a remarkably wide variation in the pKa values reported in the literature, using various methods, solvents and different ionic strengths. In aqueous solution, the values in Table 1 are reasonable estimates. Concerning monoprotonation, both phen and bpy are weaker bases than pyridine itself, with bpy being weaker than phen. This difference between phen and bpy can be explained in the additional energy required for the re-organization of the bpy from the trans- to the cis-conformation upon protonation. In contrast, for the second protonation, the [bpyH]^+^ cation is more basic than the [phenH]^+^ cation, reflecting the possibility of rotation about the inter-annular C–C bond in the [bpyH_2_]^2+^ cation to minimize charge repulsion.

### 3.4. Coordination Behaviour

The commonest coordination mode of 2,2′-bipyridine is as a chelating bidentate ligand in which both nitrogen atoms are bonded to the same metal centre. Less common are complexes in which the bpy ligand is monodentate or bridges multiple metal centres [45]. An additional coordination mode is one in which the 2,2′-bipyridine is deprotonated at C3 and the ligand functions as a C-donor or a cyclometallating C,N-donor [45]. The complexes [M(bpy)_3_]*^n^*^+^ have been intensively studied and their formation is quantified by the cumulative stability constants, *β* or *K* respectively (Equations (1)–(5), note that the solvent ligands and charges have been omitted from the equations for simplicity and that the square brackets are used in this case to indicate the equilibrium concentrations of the species rather than to enclose the coordination entity):

(1) $$M\;+\;\mathrm{bpy}\;⇌\;M(\mathrm{bpy})\;K_{1}\;=\;\beta_{1}\;=\;[M(\mathrm{bpy})]/[M][\mathrm{bpy}],$$

(2) $$M(\mathrm{bpy})\;+\;\mathrm{bpy}\;⇌\;\;M(\mathrm{bpy}{)}_{2}\;K_{2}\;=\;[M(\mathrm{bpy}{)}_{2}]/[M(\mathrm{bpy})][\mathrm{bpy}],$$

(3) $$M(\mathrm{bpy}{)}_{2}\;+\;\mathrm{bpy}\;⇌\;\;M(\mathrm{bpy}{)}_{3}\;K_{3}=\;[M(\mathrm{bpy}{)}_{3}]/[M(\mathrm{bpy}{)}_{2}][\mathrm{bpy}],$$

(4) $$M\;+\;2\mathrm{bpy}\;⇌\;\;M(\mathrm{bpy}{)}_{2}\;\beta_{2}\;=\;[M(\mathrm{bpy}{)}_{2}]/[M][\mathrm{bpy}{]}^{2},$$

(5) $$M\;+\;3\mathrm{bpy}\;⇌\;\;M(\mathrm{bpy}{)}_{3}\;\beta_{3}\;=\;[M(\mathrm{bpy}{)}_{3}]/[M][\mathrm{bpy}{]}^{3},$$

*β*_2_ = *K*_1_*K*_2_*β*_3_ = *K*_1_*K*_2_*K*_3_  lg*β*_2_ = lg *K*_1_ + lg *K*_2_  lg *β*_3_ = lg *K*_1_ + lg*K*_2_ + lg*K*_3_

In general, *K*_1_ > *K*_2_ > *K*_3_ and the complexes with 2,2′-bipyridine ligands are more stable than those with an equivalent number of pyridine ligands (i.e., comparing [M(bpy)_3_]*^n^*^+^ with [M(py)_6_]*^n^*^+^). Comparing equivalent *K* or *β* values between bpy and phen complexes reveals that the phen species are significantly more stable, with *β*_3_ showing the phen compounds to be typically 100–10,000 times more stable than their bpy analogues. This is attributed to the strain energy incurred in attaining the cis-conformation of the bpy necessary for coordination. Another way of viewing this is by describing the phen as pre-organized or pre-disposed for coordination.

## 4. The First 30 Years—from 1899 to 1929

### 4.1. Physicochemical Properties

**Figure 5:**
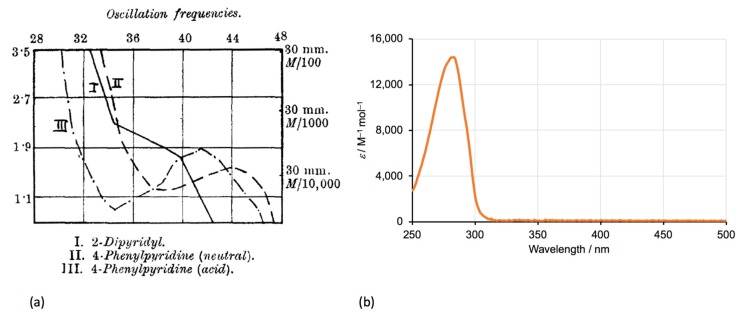
What a difference a century makes! (**a**) The first reported absorption spectrum of 2,2′-bipyridine from 1913 (Reproduced from reference 46 by permission of The Royal Society of Chemistry); (**b**) a modern absorption spectrum of the same compound (courtesy of Ms. Dalila Rocco, University of Basel). Both spectra are recorded in ethanol as solvent.

It is probably fair to say that Blau’s reports of the preparation and coordination chemistry of 2,2′-bipyridine and 1,10-phenanthroline did not set the chemical world ablaze. In view of the subsequent importance of complexes of bpy in understanding the bonding, spectroscopy, photophysics and photochemistry of metal complexes, it is perhaps appropriate that the first publication in the 20th century CE concerned the vapour phase and solution (Figure 5) absorption spectroscopy of 2,2′-bipyridine [46]. Before considering developments in the synthesis and coordination chemistry of 2,2′-bipyridine, we continue the theme of physicochemical studies. In a study of what we would now call atropisomerism of biphenyl derivatives [47], Mascarelli predicted that it might be possible to resolve some derivatives of **1** exhibiting restricted rotation and hence optical activity [48].

### 4.2. Biological Activity

**Figure 6:**
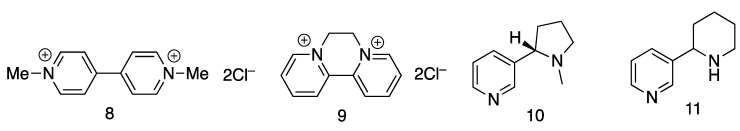
The structures of bioactive materials related to the bipyridines, the herbicides paraquat (**8**) and diquat (**9**), the alkaloid nicotine (**10**) and neonicotine (**11**). The latter compound was responsible for the reported toxicity of crude bipyridine preparations [52].

In the latter half of the 20th century CE, the quaternized derivatives **8** and **9** were commercialized as the herbicides paraquat and diquat, respectively (Figure 6) [49]. This application was predated by investigations of the biological activity of the isomeric bipyridines or their crude mixtures, in part inspired by the perceived similarity of their structures to that of the alkaloid nicotine, **10**. In a first study in 1923 of their effectiveness as contact insecticides, it was shown that impure 4,4′-bipyridine was much more toxic to *Aphis rumicis* than purified material, an observation attributed to the presence of more toxic isomeric bipyridines [50]. A subsequent publication reported that crude extracts containing the isomeric bipyridines were tested against *Aphis rumicis*, *Myzus persicae*, *Illinoia pisi*, *Rhopalosiphum pseudobrassicae*, *Anuraphis roseus*, *Aphis pomi*, *Leptinotarsa decemlineata*, *Lema trilineata*, *Ephestia kuehniella* and *Bombyx mori* [51]. The extracts were very efficient at killing aphids and studies using purified bipyridine isomers showed that all were moderately toxic, with 3,3’-bipyridine and 2,3-bipyridine being less effective than 2,2′-bipyridine or 3,4-bipyridine. However, the conclusion was that the toxicity of the crude mixture of bipyridines was less due to the bipyridines themselves, but rather to unidentified water-soluble species with an efficacy similar to nicotine. The highly toxic material was eventually identified as 3-(piperidin-2-yl)pyridine, **11**, which was given the trivial name *neonicotine* [52,53].

### 4.3. Synthetic Developments

The preparation of the bipyridines was further investigated in this period, in particular within the established mainland European laboratories, and as interest in the 2,2′-bipyridines slowly grew, new and specific synthetic methods for this isomer were developed. These fall into three main categories: (i) dimerization of a pyridine derivative with an electropositive metal and subsequent oxidation of the resultant tetrahydrobipyridine (ii) reductive coupling of a 2-functionalized pyridine with a transition metal and (iii) oxidation of a pyridine.

#### 4.3.1. Dimerization with Electropositive Metals

In Section 2 we mentioned the reaction of pyridine with sodium metal to generate blue “sodium dipyridine” and the subsequent oxidation of this to a mixture of bipyridines, predominantly the 4,4′-isomer, **3**, although it is reported that all six possible isomers are present in varying amounts [50]. Further investigations into the reaction of pyridine with sodium were reported from 1914 onwards [21,54,55,56], although these publications are primarily concerned with the preparation and reactivity of 1,1′,4,4′-tetrahydro-4,4′-bipyridines. Smith later showed that the oxidation of “sodium dipyridine” in pyridine at 90°C or lower with dry air or oxygen gave predominantly **3**, but at higher temperatures the amounts of isomeric bipyridines increased [22]. The reactions of *N*-alkylpyridinium salts or *N*-acylpyridinium salts, either from isolated compounds or prepared in situ, with reactive metals such as zinc or sodium amalgam yield 1,1’-dialkyl-1,1’,4,4′-tetrahydro-4,4′-bipyridines or 1,1’-diacyl-1,1’,4,4′-tetrahydro-4,4′-bipyridines as the primary products [57,58,59,60,61,62,63,64,65,66,67,68,69,70], although some of these were initially formulated as 2,2′-bipyridine derivatives [58].

#### 4.3.2. Reductive Coupling of a 2-Functionalized Pyridine

**Figure 7:**
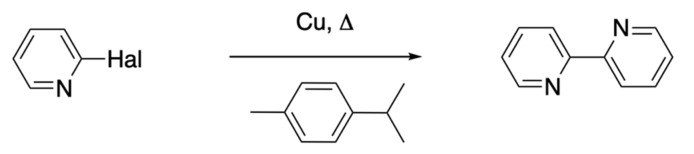
The preparation of 2,2′-bipyridine utilizing the Ullmann reaction was introduced by Wibaut in 1928 (Hal = Br or Cl) [72].

In 1928, Wibaut used the Ullmann reaction [71] for the preparation of 2,2′-bipyridine from the reaction of 2-bromopyridine or 2-chloropyridine with copper metal in 1-methyl-4-(propan-2-yl)benzene (Figure 7), obtaining **1** in 60% yield in the case of the reaction with 2-bromopyridine [72]. This reaction and modern variants remained a staple in the arsenal of synthetic routes available to the bpy chemist for many years, although the yields were variable and the work-up often “challenging”.

#### 4.3.3. Oxidation and Dehydrogenation of Pyridines and Piperidines

**Figure 8:**
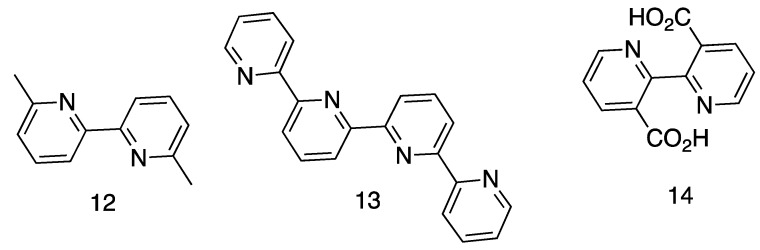
The structures of the earliest derivatives of 2,2′-bipyridine to be described; compound **12** was obtained from the pyrolysis of 2-methylpyridine [73].

Heating pyridine to a temperature between 700 °C and 800 °C in a sealed tube gives bpy as the major condensation product, together with 2,3′-bipyridine and 2,4′-bipyridine [73]; when 2-methylpyridine was treated in the same manner, 6,6′-dimethyl-2,2′-bipyridine, **12**, was isolated, making this the first substituted 2,2′-bipyridine to be prepared (Figure 8). Meyer described a red colouration when 6,6′-dimethyl-2,2′-bipyridine was treated with iron(II) salts, which suggests that his material was contaminated with bpy. Wibaut confirmed the formation of traces of bpy from the pyrolysis of pyridine [74,75] and in a closely related reaction, Sabatier showed that dehydrogenation of piperidine vapor over MnO or nickel at elevated temperatures gave a mixture of pyridine and bpy [76].

**Figure 9:**
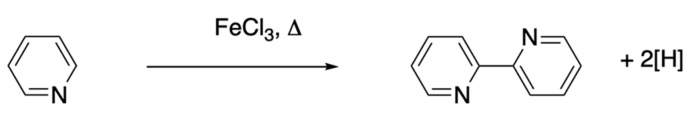
The preparation of 2,2′-bipyridine by the reaction of pyridine with FeCl_3_ at elevated temperatures provided a convenient access to the ligand in 52% yield, although the work-up was sometimes challenging because of the large amounts of metal salts [77].

Noting that the yield of 2,2′-bipyridine from the original Blau synthesis was typically below 20%, Hein also described the direct preparation of bpy from pyridine in 1928 [77]. In rationalizing his approach, he describes what we would today call a template reaction, postulating that the high stability of metal complexes of bpy, in particular of [Fe(bpy)_3_]^2+^, might be used as a driving force for the oxidative coupling of two molecules of pyridine. Pyridine was heated with FeCl_3_ at 300 °C in a sealed vessel and after (extensive) work up, 2,2′-bipyridine was obtained in a 52% yield (Figure 9). 2,2′-Bipyridine was also obtained when pyridine was heated with FeCl_3_⋅6H_2_O or CuCl_2_, but not CuCl_2_⋅2H_2_O. The colour changes in the reaction indicate reduction to iron(II) although the exact stoichiometry and, in particular, the fate of the two hydrogen atoms per molecule of bpy are not known.

### 4.4. Coordination Chemistry

**Figure 10:**
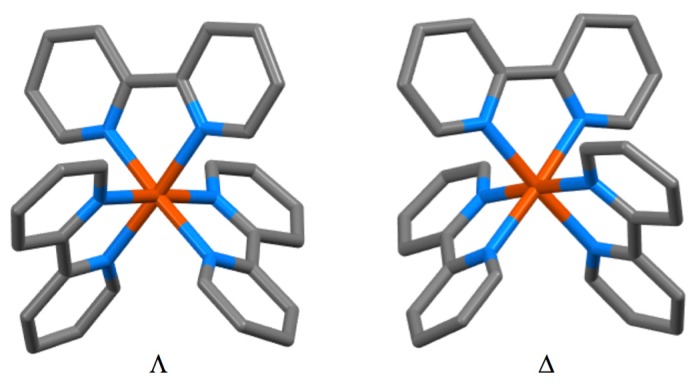
The Λ and Δ enantiomers of the [Fe(bpy)_3_]^2+^ cation resolved by Werner in 1912 [78].

It is appropriate that following the initial publications from Blau, the next studies of the coordination chemistry of bpy should be made by Alfred Werner. In 1912 [78], he described the resolution of the Λ and Δ enantiomers of the [Fe(bpy)_3_]^2+^ cation as their diastereoisomeric l-tartrate salts (Figure 10). Werner described the rapid racemization of the [Fe(bpy)_3_]^2+^ cation and postulated that it might be due to the dissociation of bpy from the complex. The next chemical study of the coordination behaviour dates to 1926 and is concerned with the adsorption of [Fe(bpy)_3_]^2+^ and [Fe(phen)_3_]^2+^ on various substrates, including blood charcoal (*Blutkohle*) and natural polymers such as wool [79]. The chiral nature of wool and other natural polymers was specifically mentioned but no diastereoselectivity was observed in the binding of the complex cations. The authors also conclude that dissociation of bpy from [Fe(bpy)_3_]^2+^ occurs under specific conditions and conclude inter alia “the sulfate of ferro-tridipyridyl and the bromide of ferro-triphenantroline, are strongly adsorbed in aqueous solution by adsorbents such as charcoal, wool, arsenic trisulfide....An aqueous solution...that initially wets glass and quartz well, only wets glass and quartz very poorly after a short time; it appears that a poorly wetting organic substance is formed....probably due to the separation of dipyridyl from the complex salts”.

Today, we describe bpy as a chelating ligand and the coordination compounds as chelate complexes, but this description was only introduced in 1920 when Morgan and Drew [80] used the word chelate to describe a ligand which bound to a metal ion through two different atoms. Chelated complexes of bpy and phen were cited in a bad-tempered exchange between Lowry and Smith in 1923 debating the need for the term and regarding the precise manner which the term chelate should be used [81,82,83].

The year 1928 saw the first magnetochemical investigation of a coordination compound containing bpy, when Blitz reported that the salt [Fe(bpy)_3_](SO_4_) was diamagnetic and, in modern terminology, a low spin iron(II) complex [84].

## 5. 1930–1939—Golden Years and then Back into the Abyss

In this next section, the developments in the period 1930–1939 are reviewed. The principal advances came in the elaboration of the coordination chemistry of bpy and related ligands, in particular the pioneering studies of Morgan and Jaeger. In parallel, synthetic methods for the bipyridines were being refined and, increasingly, the ligands and their complexes were being investigated in physicochemical and analytical applications.

### 5.1. Synthetic Developments

This section is concerned primarily with synthetic studies leading to bpy and its derivatives.

#### 5.1.1. Oxidation and Dehydrogenation of Pyridines

Hein and Retter had shown in 1928 that acceptable yields of bpy could be obtained from the reaction of pyridine with FeCl_3_ or other metal salts [77] and this method of preparation proved to be something of a growth industry in the 1930s and seems to have become the preparative method of choice for the coordination chemists [85,86]. On a personal note from one of the authors (E.C.C.), these methods should be avoided if possible, as the work-up of the dark-coloured and foul-smelling reaction mixture is truly disgusting! Morgan showed that not only 2,2′-bipyridine was formed in this reaction, but also higher oligopyridines, in particular 1^2^,2^2^:2^6^,3^2^-terpyridine (2,2′:6′,2″-terpyridine, **7**) and 1^2^,2^2^:2^6^,3^2^:3^6^,4^2^-quaterpyridine (2,2′:6′,2″:6″,2‴-quaterpyridine, **13**, Figure 8) [87,88]. Heating pyridine with WCl_6_, [Cu(py)_2_Cl_2_], [Co(py)_4_Cl_2_] or (Hpy)[FeCl_4_] gave moderate to good yields of 2,2′-bipyridine and other isomeric bipyridines and higher oligopyridines [88]. The use of the copper and cobalt complexes raises again the question of whether these are template reactions, with the critical interannular C–C bond formation involving coordinated pyridines. Non-metallic oxidizing agents are also effective for the conversion of pyridine to bpy and heating pyridine with I_2_ at 320 °C gave good yields of bpy [89]. Traces of bpy were also obtained by heating pyridine alone or with dihydrogen on the presence of catalytic amounts of AlCl_3_ and FeCl_3_ [90]. Burstall subsequently showed that the reaction of 2-methylpyridine with FeCl_3_ could be used for the preparation of 6,6’-dimethyl-2,2′-bipyridine, **12** (Figure 8) [91].

Wibaut showed that respectable yields of bpy could be obtained from the dehydrogenation of pyridine at 300 °C with Ni-metal catalysts [92]. The same catalysts were used by Willink for the conversion of 2-methylpyridine to **12**, which was reported not to give a red-colouration with iron(II) salts [89]. In mechanistically obscure reactions, Wibaut also showed that the yield of bpy from the reaction of pyridine with dehydrogenation catalysts at elevated temperatures was increased in the presence of ammonia [93]. This method was extended to the preparation of 6,6’-dimethyl-2,2′-bipyridine from 2-methylpyridine, albeit in low yield, by Burstall [91].

The isolation of bpy as a minor product in the reaction of pyridine with NaNH_2_ is explained in terms of a metallation reaction competing with the nucleophilic attack of amide, followed by subsequent reaction of the metallated intermediate with pyridine and oxidation of the resultant dihydro-2,2′-bipyridine in the workup [94]. The reaction of sodamide with bpy itself gives 6,6′-diamino-2,2′-bipyridine in low yield [89]

#### 5.1.2. Other Routes to 2,2′-Bipyridines

**Figure 11:**
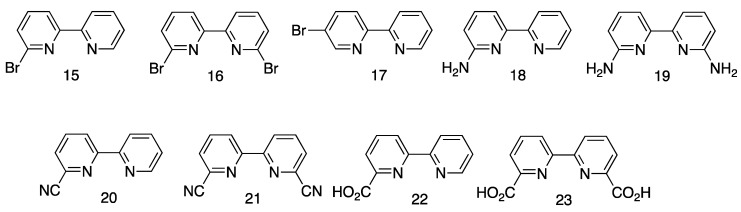
The structures of some early derivatives of 2,2′-bipyridine.

The second derivative of 2,2′-bipyridine to be described was 2,2′-bipyridine-3,3’-dicarboxylic acid **14** (Figure 8) which was obtained from the oxidation of 1,10-phenanthroline [89,95]. In 1938, Burstall gave an overview of the methods available for the preparation of bpy and reported the use of diphenyl as a solvent for the preparation of higher oligopyridines by Ullmann reactions [91]. In the course of these studies, Burstall described the preparation of both 6-bromo-2,2′-bipyridine, **15**, and 6,6’-dibromo-2,2′-bipyridine, **16** (Figure 11), from the vapour-phase bromination of bpy at 773 K. At lower temperatures (523 K), only a perbromide was obtained and no brominated-2,2′-bipyridines could be isolated. In contrast, the reaction of (bpyH)Br with bromine at 523 K gave a mixture of 6-bromo-2,2′-bipyridine and 5-bromo-2,2′-bipyridine, 17. The relevant bromo-compounds were converted to other bpy derivatives, for example 6-amino-2,2′-bipyridine, **18**, and 6,6’-diamino-2,2′-bipyridine, **19** (Figure 11), by reaction with aqueous ammonia at > 473 K or 2,2′-bipyridine-6-carbonitrile, **20**, and 2,2′-bipyridine-6,6’-bis(carbonitrile), **21**, by reaction with CuCN. The carboxylic acids, 2,2′-bipyridine-6-carboxylic acid, **22**, and 2,2′-bipyridine-6,6’-dicarboxylic acid, **23**, were obtained by the hydrolysis of the corresponding nitriles with concentrated HCl, although the latter compound was also obtained from the oxidation of 6,6’-dimethyl-2,2′-bipyridine, **12**, with selenium dioxide.

### 5.2. Coordination Chemistry

The 1930s saw the growth in the coordination chemistry of bpy, in part inspired by the development of better synthetic methods for the ligand, and in part by a general increase of interest in inorganic and coordination chemistry. In the period up to 1930, the best characterized complexes exhibited a 3:1 bpy:M ratio, typified by salts of [Fe(bpy)_3_]^2+^, although Blau had also described 1:1 and 2:1 complexes with copper(II) [29]. In the 1930’s, the broad picture of the diversity of complexes formed by bpy across the entire periodic table emerged, in particular with the body of work for Jaeger and Morgan and Burstall. Jaeger was particularly important in making extensive use of crystallographic studies (but not single crystal determinations) in his work. One feature that emerges from this early work is that many of the compounds reported and apparently exhibiting simple stoichiometries, were subsequently shown to exhibit structural complexity or to possess unexpected reactivity not suspected at the initial time of the preparation.

#### 5.2.1. Complexes of Elements in Groups 1 and 2

No complexes of elements of group 1 and 2 containing bpy were reported in the 1930s (in contrast to pioneering descriptions of complexes of phen with these elements).

#### 5.2.2. Complexes of Elements in Groups 3 to 12

Considering the early history of bpy, it is not surprising that the vast majority of publications concerning its coordination chemistry reported in the period up to 1939 concerned iron(II) complexes. The most important development was probably the use of bpy and phen as reagents for the colorimetric determination of iron in a wide range of materials for which efficient and effective methods had not previously been available. These studies are collected in Section 5.3. However, other studies of bpy complexes with group 8 metals were also reported in this period.

**Figure 12:**
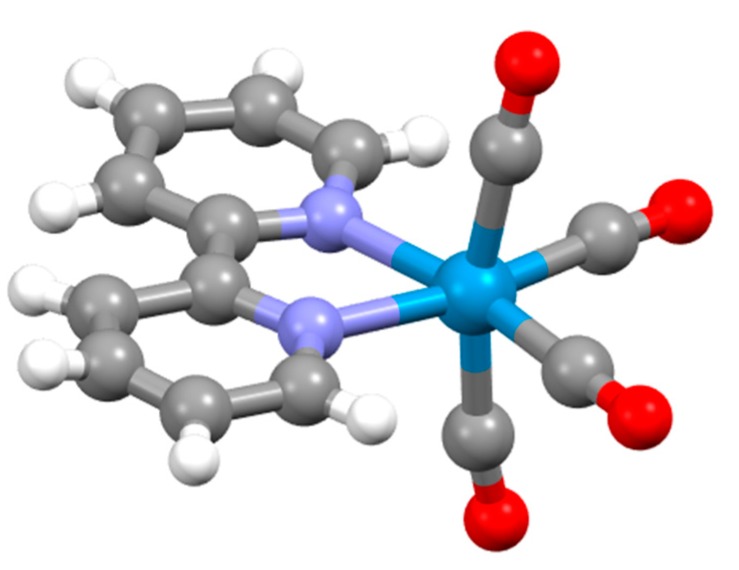
The solid-state structure of [W(bpy)(CO)_4_] (structural data taken from reference [102].

No complexes with elements of groups 3–5 were reported. The first group 6 compound was probably described when Blau mentioned the formation of a red solution as bpy reacted with chromium(III) solutions, but he made no further characterization of the products [29]. The first characterized chromium complex was [Cr(bpy)_3_]Br_2_.6H_2_O, which was prepared by the reaction of [Cr_2_(OAc)_4_] with (bpyH)Br; the compound is relatively air-stable in aqueous solution [96,97]. Although the formation of coloured species when low oxidation state molybdenum reacted with bpy, no complexes were isolated [98,99]. The first mixed ligand complex containing bpy and carbonyl ligands was reported by Hieber in 1935, who obtained dark-red [W(bpy)(CO)_4_] from the reaction of [W(CO)_6_] with bpy in EtOH [100]. The compound [W(bpy)(CO)_4_] has been structurally characterized and has the structure predicted by Hieber (Figure 11 and Figure 12) [101,102]. Unexpected is the fact that the compound crystallizes in the non-centrosymmetric space group *Pn* (No. 7) and is an effective material for second harmonic generation (similar to urea) [101].

Although no manganese complexes were reported in this early period, a detailed study of rhenium chemistry was reported by Morgan [103]. No complexes with bpy ligands coordinated to rhenium were isolated, but the salts (bpyH_2_)[ReO_2_(CN)_4_]_2_ (pink), H(bpyH)[ReO_2_(CN)_4_] (blue), (bpyH)_2_[ReCl_6_] (green), (bpyH_2_)[ReCl_6_] (yellow) and (bpyH)[ReO_4_] (colourless) were described. Subsequently, the compounds (bpyH_2_)[ReCl_6_] [104] and (bpyH)_2_[ReCl_6_] [104,105] have been structurally characterized.

The elements of group 8 have attracted the most attention and it is not an understatement to say that in this period, the 3:1 bpy:Fe complex dominates the chemistry of bpy. Most researchers working actively on bpy and its derivatives recognize with horror the pale pink colour that often pervades their reaction mixtures. The stability of the [Fe(bpy)_3_]^2+^ cation particularly intrigued Italian researchers. In a note on the analytical applications of bpy, Ferrari commented on the extreme stability of [Fe(bpy)_3_]^2+^, noting that it does not give a blue colour with K_3_[Fe(CN)_6_] and that the iron is not precipitated by aqueous ammonia solution [106]. He also noted that in aqueous solution, regardless of the Fe:bpy ratio, the only iron(II)-bpy species present was [Fe(bpy)_3_]^2+^ [107]. In contrast, Barbieri showed that in acidic solution at elevated temperatures, the complex decomposed to give (bpyH_2_)^2+^ and [Fe(H_2_O)_6_]^2+^, but regenerated [Fe(bpy)_3_]^2+^ on cooling [108]. Jaeger described materials with apparently 1:1, 2:1 and 3:1 bpy:Fe ratios from the reaction of bpy with FeSO_4_, although metathesis to chloride salts with BaCl_2_ gave [Fe(bpy)_3_]Cl_2_ in all cases [109]. Magnetic studies of a series of bpy complexes were reported and the tendency for the bidentate N,N-donor to form low-spin complexes was confirmed [110,111,112,113] although high-spin complexes with thiocyanato ligands were described for the first time in 1934 [114].

**Figure 13:**
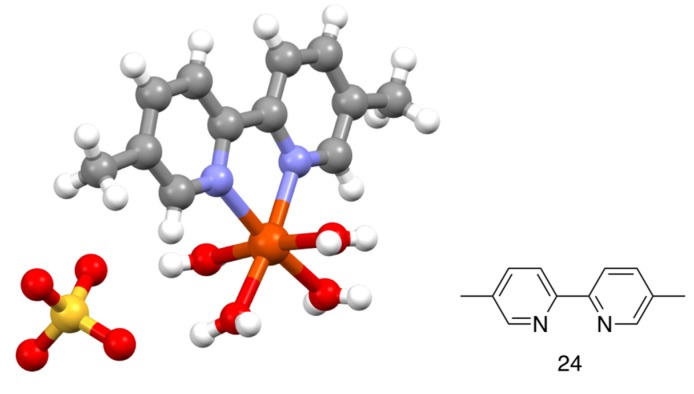
The solid-state structure of [Fe(**24**)(H_2_O)_4_](SO_4_) (structural data taken from reference 117) and the structure of 5,5′-dimethyl-2,2′-bipyridine, **24**.

In 1934, Barbieri reported the preparation of diamagnetic complexes [Fe(bpy)_2_(CN)_2_] (red-violet) and [Fe(bpy)(CN)_4_]^2–^ (isolated as the orange-yellow K^+^, Ba^2+^, Pb^2+^ and Ag^+^ salts as well as the free acid) by heating [Fe(bpy)_3_]^2+^ salts with aqueous KCN solution [115]. The analogous compound [Fe(bpy)_2_(SCN)_2_] was described by Ferrari [107]. Jaeger also described the isolation and crystal morphology of a series of complexes containing iron and bpy, including [Fe(bpy)_3_]Cl_2_.7H_2_O and {Fe(bpy)(H_2_O)_2.33_(SO_4_)} [116]. The nature of these 1:1 and 1:2 complexes [109,116] remains unclear, but it is worth noting that the complex [Fe(**24**)(H_2_O)_4_](SO_4_) (**24** = 5,5′- dimethyl-2,2′-bipyridine, Figure 13) has been structurally characterized (Figure 13) [117].

In an early study of homogeneous catalysis, it was noted that iron(III) in the presence of bpy oxidizes H_2_O_2_ with the formation of [Fe(bpy)_3_]^2+^ [118,119,120]; in the course of the reaction, until the complete formation of the complex cation, species are present which are highly active for the catalytic decomposition of H_2_O_2_. These reactions and an understanding of their mechanism are still relevant today for the myriad of poorly understood reactions involving water, light and bpy complexes. The use of bpy to determine iron(II) concentrations in the oxidation of iron(II) to iron(III) by H_2_O_2_ should be assessed cautiously if intermediate Fe-bpy complexes are highly catalytically active [121].

**Figure 14:**
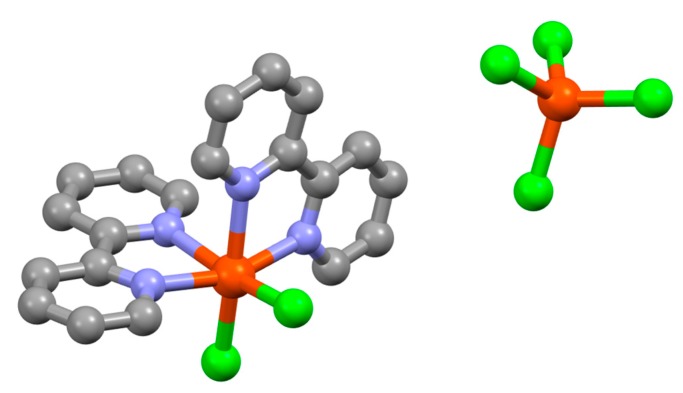
The solid-state structure of structure of {Fe(bpy)Cl_3_}, which was shown to be [Fe(bpy)_2_Cl_2_][FeCl_4_] (structural data taken from reference 129).

Few attempts to prepare iron(III) complexes were reported, but of note is the synthesis of {Fe(bpy)Cl_3_} from the reaction of FeCl_3_ with bpy in ether [122]. The complex is a high-spin iron(III) species with μ_eff_ of 5.91 B.M.. It has subsequently been shown that six coordinate mononuclear species with an additional ligand, [Fe(bpy)XCl_3_] (X = H_2_O [123], MeOH [123,124], 1,2,4-triazole [125], dmso [126]) are relatively common, but the structure of the parent compound is more complex. Multiple structure determinations of different polymorphs of {Fe(bpy)Cl_3_} have shown that in the solid state it is not five-coordinate but correctly formulated as [Fe(bpy)_2_Cl_2_][FeCl_4_] (Figure 14) [127,128,129,130]. The reaction of [Fe(bpy)_2_Cl_2_][FeCl_4_] with H_2_S was also described, which proceeded according to the stoichiometry of Equation (6).

6Fe(bpy)Cl_3_ + 3H_2_S → 2[Fe(bpy)_3_]Cl_2_ + 4FeCl_2_ + 6HCl + 3S, (6)

In view of the dominant role that ruthenium, and to a lesser extent osmium, complexes containing bpy-type ligands were to play in the latter half of the 20th Century CE, it is perhaps surprising how little attention these elements received in this golden age of coordination chemistry. The first mention of ruthenium bpy complexes occurs in a published lecture from Morgan dating to 1935 [131] although the details of these new compounds were only published in 1938. The first report of [Ru(bpy)_3_]^2+^ was made in 1936, when Burstall described the reaction of ruthenium trichloride with bpy according to Equation (7) [132]. The reduction of ruthenium(II) (or strictly higher oxidation states of ruthenium, as “ruthenium trichloride” is a complex material) to ruthenium(II) in orange [Ru(bpy)_3_]^2+^ was accompanied by the oxidation of bpy to 1^2^,2^2^:2^6^,3^2^:3^6^,4^2^-quaterpyridine (**13**, Figure 8).

2RuC1_3_ + 8bpy → 2[Ru(bpy)_3_]Cl_2_ + **13** + 2HCl (7)

**Figure 15:**
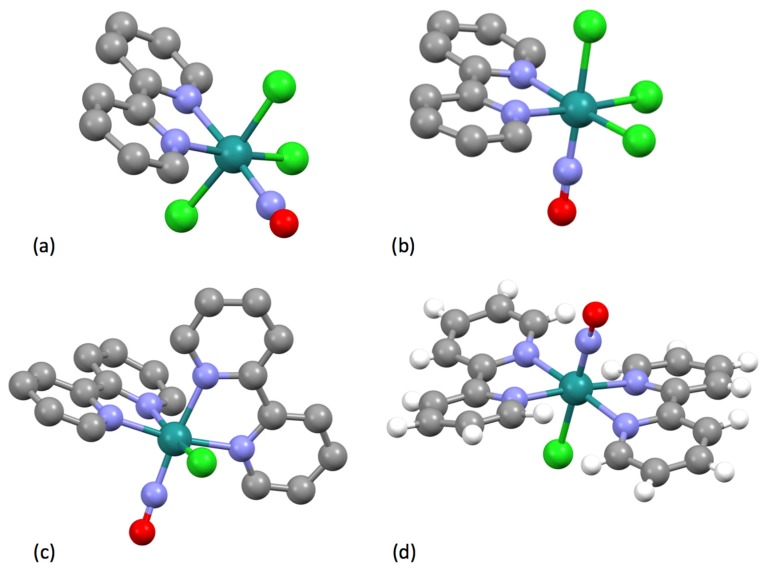
(**a**) *mer*-[Ru(bpy)Cl_3_(NO)] [134] (**b**) *fac*-[Ru(bpy)Cl_3_(NO)] [134] (**c**) the *cis*-[Ru(bpy)_2_Cl(NO)]^2+^ cation in [Ru(bpy)_2_Cl(NO)](ClO_4_)_2_ [135] (**d**) the *trans*-[Ru(bpy)_2_Cl(NO)]^2+^ cation in [Ru(bpy)_2_Cl(NO)](ClO_4_)_2_ [136].

Burstall notes that the complex ion [Ru(bpy)_3_]^2+^ is significantly more stable than the iron(II) and nickel(II) analogues and describes the resolution into the Λ and Δ salts with d-tartrate. Burstall also notes the remarkable kinetic stability of the enantiomeric cations and reports that not only do the salts show no racemization in aqueous solution at room temperature but also upon boiling, in contrast to the iron and nickel homologues. Burstall also described the preparation of [Ru(bpy)_3_]^2+^ salts from the reaction of ruthenium trichloride with bpy in aqueous or ethanolic solution. He also obtained the same product from the reaction of “ruthenium red” (probably [(NH_3_)_5_RuORu(NH_3_)_4_ORu(NH_3_)_5_]Cl_6_ but at the time formulated [RuCl(NH_3_)_4_(OH)]Cl.H_2_O) or K[Ru(NO)Cl_5_] with bpy at 250 °C or in aqueous solution or from the reaction of a variety of ruthenium-chlorido complexes in various oxidation states with bpy. In 1938, Morgan and Burstall described a series of ruthenium-nitroso complexes, including [Ru(bpy)Cl_3_(NO)], [Ru(bpy)Br_3_(NO)], [Ru(bpy)I_3_(NO)], [Ru(bpy)_2_Cl(NO)][RuCl_5_(NO)] and (bpyH)_2_[RuCl_5_(NO)] [133]. Both *mer*- (Figure 15 a) and *fac*- (Figure 15b) diastereoisomers of *fac*-[Ru(bpy)Cl_3_(NO)] have been structurally characterized; as expected the nitrosyl ligands are linear [134]. Although [Ru(bpy)_2_Cl(NO)][RuCl_5_(NO)] has not been structurally characterized, the structure of both *cis*- (Figure 15c) [135] and *trans*- (Figure 15d) [136] diastereoisomers of the [Ru(bpy)_2_Cl(NO)]^2+^ cation in the related salt [Ru(bpy)_2_Cl(NO)](ClO_4_)_2_ have been described. The latter cation is a relatively rare example of an [M(bpy)_2_XY]*^n^*^+–^ complex exhibiting a trans-arrangement of the X and Y ligands and shows the riffling of the bpy ligands that is necessary to minimize the bpy H6…H6 interactions.

**Figure 16:**
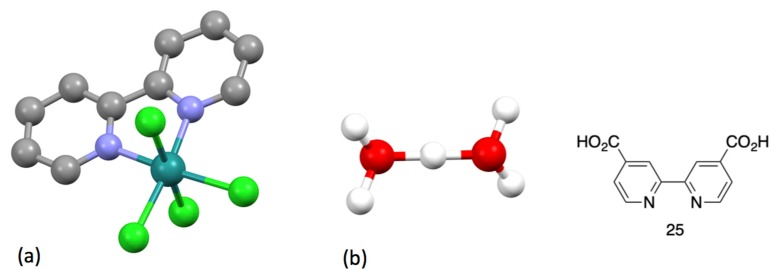
(**a**) The structure of the [Ru(bpy)Cl_4_]^–^ anion in K[Ru(bpy)Cl_4_] [138] and (**b**) the (H_5_O_2_)^+^ cation present in [(H_5_O_2_)Ru(**25**)Cl_4_].2H_2_O (**25** = 2,2′-bipyridine-4,4′-dicarboxylic acid) [139].

Goodward reported his investigations on the notorious “ruthenium blue” solutions obtained by the reduction of ruthenium compounds in various solvents. He used a blue solution obtained by the electrochemical reduction of ruthenium trichloride in 6M hydrochloric acid and isolated a compound he formulated as variously as {(bpyH)RuCl_4_} or {(bpyH_2_)RuCl_4_} [137]. This compound is probably the acid H[Ru(bpy)Cl_4_]; the salt K[Ru(bpy)Cl_4_] has been structurally characterized (Figure 16a) [138] as has the related compound [(H_5_O_2_)Ru(**25**)Cl_4_].2H_2_O (**25** = 2,2′-bipyridine-4,4′-dicarboxylic acid) containing the unusual (H_5_O_2_)^+^ cation (Figure 16b) [139]. The true complexity of the ruthenium blue solutions has been addressed by Seddon [140] but the conversion of the blue solution to [Ru(bpy)_3_]^2+^ and other bpy complexes lay in the distant future.

**Figure 17:**
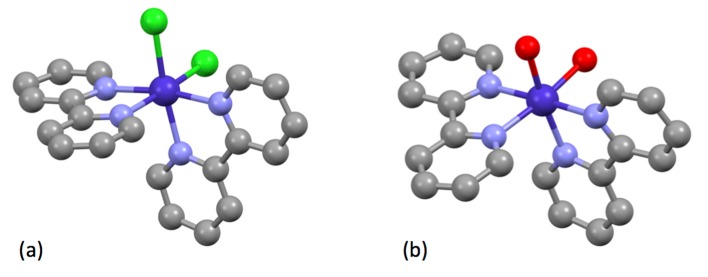
(**a**) The neutral cobalt(II) coordination entity present in [Co(bpy)_2_Cl_2_].3H_2_O [141] (**b**) the [Co(bpy)_2_(H_2_O)_2_]^2+^ cation present in [Co(bpy)_2_(H_2_O)_2_][{N(CH_2_CO_2_)_3_}Cr(μ-OH)Cr{N(CH_2_CO_2_)_3_] [143].

The group 9 metals were to play a vital role in the development of bpy chemistry, and the eventual recognition of the cyclometallated C,N-bonding mode was to be established in iridium complexes. In studies of the coordination chemistry of cobalt with bpy, Jaeger described [Co(bpy)_3_]Cl_2_.6H_2_O, [Co(bpy)_2_Cl_2_].5H_2_O, [Co(bpy)_2_(H_2_O)_2_]Cl_2_.4H_2_O, [Co(bpy)_2_(H_2_O)_2_]Cl_2_.3H_2_O [Co(bpy)_3_]SO_4_.7H_2_O, [Co(bpy)_2_(H_2_O)_2_]SO_4_.4H_2_O, [Co(bpy)_3_](NO_3_)_2_.6H_2_O, [Co(bpy)_2_CO_3_]NO_3_.5H_2_O, [Co(bpy)_2_Cl_2_]Cl.2H_2_O, [Co(bpy)_3_]_2_(SO_4_)_3_.5H_2_O and [Co(bpy)_3_]Cl_3_.3H_2_O [116,141]. Jaeger was remarkably prescient in his assignment of the coordination mode in these compounds, an especially difficult task with labile metal centres such as cobalt(II) and in the absence of the modern analytical methods that we take for granted. Although [Co(bpy)_2_Cl_2_].5H_2_O has not been structurally characterized, the trihydrate has (Figure 17a), confirming the presence of two chlorido ligands coordinated to the cobalt(II) centre [142]. The cationic [Co(bpy)_2_(H_2_O)_2_]^2+^ coordination entity has been established in the compound [Co(bpy)_2_(H_2_O)_2_][{N(CH_2_CO_2_)_3_}Cr(μ-OH)Cr{N(CH_2_CO_2_)_3_] (Figure 17b) [143]. The structural analysis of green [Co(bpy)_2_Cl_2_]Cl.2H_2_O confirmed the presence of the CoN_4_Cl_2_ coordination core with one free anionic chloride ion [144,145]. Finally, structural analyses of [Co(bpy)_2_CO_3_]NO_3_.5H_2_O confirmed the presence of the coordinated bidentate carbonato ligand and ionic nitrate, rather than alternative structures with coordinated nitrato ligands [146,147].

As early as 1934, Jaeger described the preparation and crystal morphology of the archetypical complexes [Rh(bpy)_3_]Cl_3_.3H_2_O, [Rh(bpy)_2_Cl_2_]Cl.2H_2_O, [(bpy)_2_Rh(μ-Cl)_2_Rh(bpy)_2_]Cl_4_ together with the aqua-species [Rh(bpy)(H_2_O)_4_]^3+^, [Rh(bpy)(H_2_O)_2_Cl_2_]^+^, [Rh(bpy)(H_2_O)_3_Cl]^2+^ and [Rh(bpy)(H_2_O)Cl_3_].H_2_O [116,148]. A crystal structure determination of [Rh(bpy)_2_Cl_2_]Cl.2H_2_O confirmed the presence and stereochemistry of the expected *cis*-[Rh(bpy)_2_Cl_2_]^+^ cation [149] although no structures of the aqua complexes have been reported. The compound [(bpy)_2_Rh(μ-Cl)_2_Rh(bpy)_2_]Cl_4_ is of particular interest as each metal is a stereogenic centre and the dication could exist as a number of diastereoisomers: the enantiomeric pair ΔΔ- and ΛΛ-[(bpy)_2_Rh(μ-Cl)_2_Rh(bpy)_2_]^4+^ and the diastereoisomer ΔΛ-[(bpy)_2_Rh(μ-Cl)_2_Rh(bpy)_2_]^4+^.

**Figure 18:**
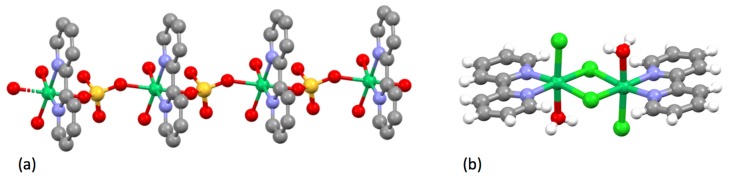
(**a**) The one-dimensional coordination polymer present in {Ni(bpy)(H_2_O)_2_(SO_4_)}.*n*H_2_O [153] and (**b**) the dimer [(bpy)Cl(H_2_O)Ni(μ-Cl)_2_Ni(bpy)Cl(OH_2_)] [160].

Blau had described the first group 10 metal complexes of bpy, the pink [Ni(bpy)_3_]^2+^ salts, in 1898 [29]. In 1931, Morgan and Burstall showed that this cation could be resolved into diastereoisomeric Λ and Δ d-tartrate salts [150,151], making this the second [M(bpy)_3_]^2+^ and (probably) the first nickel(II) complex to be resolved [78]. In the first systematic study of [M(bpy)_2_X_2_] compounds, Pfeiffer showed that when [Ni(bpy)_3_]Cl_2_ was heated over boiling benzene, toluene or xylenes, bpy was lost with a corresponding change in colour from red-brown to green and the formation of [Ni(bpy)_2_Cl_2_] [152]. Jaeger reported the synthesis and described the crystal forms of red [Ni(bpy)_3_]^2+^ salts, but also described the formation of blue materials of composition {Ni(bpy)(H_2_O)_6_(SO_4_)}, {Ni(bpy)_2_(H_2_O)_7_(SO_4_)} [86,116]. The compound {Ni(bpy)(H_2_O)_6_(SO_4_)} has been shown to be a one-dimensional coordination polymer with bridging sulfato ligands (Figure 18a) [153]. The speciation is not so simple, as the mononuclear complex [Ni(bpy)(H_2_O)_4_](SO_4_) has also been structurally characterized [154]. Pfeiffer prepared a series of [Ni(bpy)_3_]^2+^ salts in the context of his studies of what later became termed the Pfeiffer effect (see later), but does not appear to have reported their behaviour in detail [155]. Jaeger also initially confirmed the observation of Pfeiffer, that 2:1 bpy:Ni complexes could not be prepared directly in aqueous solution [86]. In later work, Jaeger showed that the reaction of nickel(II) nitrate with bpy in aqueous solution could give [Ni(bpy)_3_](NO_3_)_2_.5H_2_O, {Ni(bpy)_2_(NO_3_)_2_(H_2_O)_3_} and {Ni(bpy)(H_2_O)_3_(NO_3_)_2_} [116,156]. The nitrates are of some interest. When the reaction of bpy with nickel(II) nitrate is performed in an ionic liquid or MeOH, the complex [Ni(bpy)_2_(ONO_2_)](NO_3_) is obtained, containing a bidentate O,O′-donor nitrato ligand and a nitrate anion [157,158]. In aqueous solution, the complex [Ni(bpy)_2_(H_2_O)(ONO_2_)](NO_3_) has been structurally characterized [159], as has Jaeger′s {Ni(bpy)(H_2_O)_3_(NO_3_)_2_}, which was shown to be [Ni(bpy)(H_2_O)_3_(ONO_2_)](NO_3_) [157]. In similar studies with nickel(II) chloride in aqueous solution, Jaeger obtained [Ni(bpy)_3_]Cl_2_.7H_2_O although this yielded {Ni(bpy)_2_Cl_2_} upon heating. In reactions with one equivalent of bpy, a compound {Ni(bpy)(H_2_O)_2_Cl_2_} was obtained after the precipitation of [Ni(bpy)_3_]Cl_2_.7H_2_O. Although {Ni(bpy)(H_2_O)_2_Cl_2_} has not been structurally characterized, the related compound [(bpy)Cl(H_2_O)Ni(μ-Cl)_2_Ni(bpy)Cl(OH_2_)], with *trans*-chlorido and aqua ligands (Figure 18b), is obtained from the reaction of NiCl_2_.6H_2_O with bpy in DMSO and has a very short (3.441 Å) Ni...Ni contact [160]. Similarly, {Ni(bpy)_2_Cl_2_} has not been structurally characterized, but the cation [Ni(bpy)_2_Cl(OH_2_)]^+^ has been shown to be present in [Ni(bpy)_2_Cl(OH_2_)]Cl.CHCl_3_.H_2_O [161]; in contrast, the five-coordinate [Ni(bpy)_2_Cl]^+^ ion is present in [Ni(bpy)_2_Cl](NO_3_).3H_2_O [162]. The magnetic properties of [Ni(bpy)_3_]Br_2_.3H_2_O were first reported by Cambi in 1934 [163].

As a part of the classical series of papers from Chatt and Mann describing studies on the structure of palladium-phosphane complexes, the preparation of both [Pd(bpy)(NO_2_)_2_] and [Pd(bpy)Cl_2_] were reported [164,165]. The compound [Pd(bpy)Cl_2_] exists in two polymorphs corresponding to the red [166] and yellow [167] forms of [Pt(bpy)Cl_2_] (see below) with Pd…Pd separations of 3.46 and 4.587 Å respectively. Solvates with CH_2_Cl_2_ [168] and dioxane [169] have been structurally characterized and shown not to exhibit short Pd…Pd contacts.

**Figure 19:**
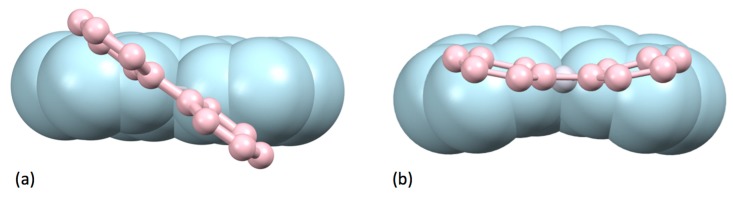
The [Pt(bpy)_2_]^2+^ is distorted from the idealized square-planar geometry with the bpy ligands lying in the square-plane because of unfavourable steric interactions between H6 protons of the bpy ligands. The complexes are either distorted to (**a**) tetrahedral arrangement of the nitrogen donors about the platinum or (**b**) the ligands are riffled about a square-planar metal centre [179].

The first reports of platinum bpy complexes were made in 1933 by Morgan and Burstall [88] and almost simultaneously, Rosenblatt found that although yellow [Pt(bpy)Cl_2_] could be prepared with ease, but reported that it was not possible to obtain [Pt(bpy)_2_]^2+^ salts in solution by reaction of K_2_[PtCl_4_] or [Pt(bpy)Cl_2_] with excess bpy in aqueous, ethanolic or phenol solution [170]. He also reported that although [Pt(bpy)Cl_2_] did not react with ammonia or pyridine, the chlorido ligands could be replaced by ethane-1,2-diamine (en) to give [Pt(bpy)(en)]^2+^ salts, which in turn reacted with KCN to give both yellow and red forms of [Pt(bpy)(CN)_2_] [170]. It is now known that in [Pt(bpy)_2_]^2+^ salts, steric interactions between the bpy H6 protons are expected in a planar structure. Nevertheless, a number of [Pt(bpy)_2_]^2+^ salts have been prepared and structurally characterized and the ways in which the complexes adapt to minimise the steric interactions are of considerable interest. In some cases, the electronic demands of the bpy ligand to maximize conjugation in a planar conformation dominates and the geometry of the PtN_4_ motif in the [Pt(bpy)_2_]^2+^ cation is distorted towards tetrahedral, with least squares planes angles between the near-planar bpy ligands between 30 and 37° (Figure 19a) [171,172,173]. In other cases, the electronic preference of the d^8^ metal centre for a square-planar PtN_4_ motif dominates, and the bpy adopts a V-shaped conformation about the interannular C–C bond (Figure 19b) [174,175,176,177,178,179]. Only in the compound [Pt(bpy)_2_](ClO_4_)_2_ have both the square-planar and tetrahedral PtN_4_ coordination geometries been structurally characterized. The [Pd(bpy)_2_]^2+^ cation shows a similar pattern of distortions [180,181,182,183,184,185,186,187,188,189].

**Figure 20:**
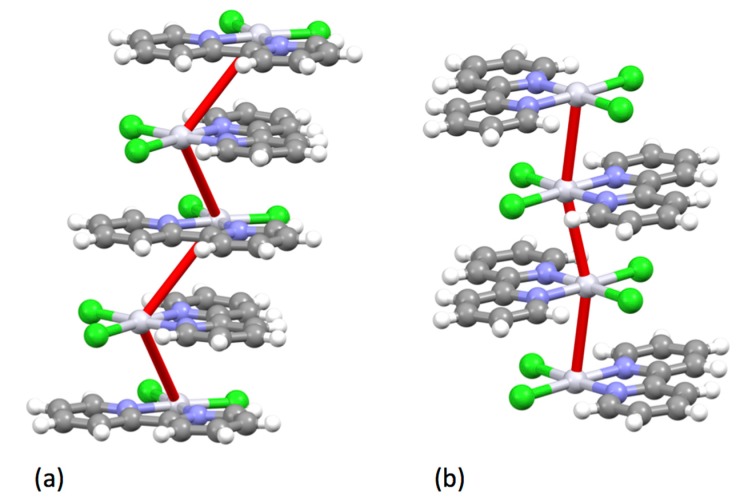
(**a**) The yellow polymorph [Pt(bpy)Cl_2_] contains zig-zag chains of Pt centres while (**b**) in the red polymorph the metal centres are closer and the propagation along the Pt...Pt...Pt vector is closer to linear.

It was left to Morgan and Burstall to further elucidate the chemistry of the platinum compounds [190]. These authors described the yellow (β) and red (α) polymorphs of [Pt(bpy)Cl_2_] and reproducible methods for their preparation. In a very early study of the photophysical properties of transition metal complexes, Randall reported the low temperature fluorescence of both the red and the yellow polymorphs [191]. Both the red [192,193,194] and the yellow [167,192,193,195,196] polymorphs of [Pt(bpy)Cl_2_] have been structurally characterized; in the yellow form, stacked planar [Pt(bpy)Cl_2_] molecules are arranged to give a zig-zag chain of Pt centres (∠Pt...Pt...Pt, 110.25°, Pt...Pt 4.56 Å) whereas in the red form, the metal centres are much closer together (Pt...Pt 3.45 Å) and the propagation along the Pt...Pt...Pt vector is closer to linear (∠Pt...Pt...Pt, 161.2°) Figure 20.

Morgan and Burstall also demonstrated the formation of [Pt(bpy)_2_]^2+^ salts and suggested that some of the {Pt(bpy)Cl_2_} materials reported by Rosenblatt in his attempts to prepare [Pt(bpy)_2_]^2+^ salts, might be the coordination isomer [Pt(bpy)_2_][PtCl_4_]. In addition to confirming the [Pt(bpy)(en)]^2+^ species, compounds containing the [Pt(bpy)(NH_3_)_2_]^2+^, [Pt(bpy)(py)_2_]^2+^, [Pt(bpy)(py)Cl]^+^, [Pt(bpy)(NH_3_)Cl]^+^, [Pt(bpy)(NH_3_)I]^+^ and [Pt(bpy)(MeSCH_2_CH_2_SMe)]^2+^ cations were also prepared. Salts of [Pt(bpy)(en)]^2+^ and related compounds have attracted some interest because of their ability to exhibit strong face-to-face π-interactions with aromatics (in particular nucleic acids), and a number of compounds have been structurally characterized [197,198,199]. In [Pt(bpy)(py)_2_]^2+^ salts, the pyridine ligands are close to orthogonal to the PtN_4_ square plane containing the bpy ligand [200,201,202]. Finally, Morgan and Burstall extended the chemistry to the platinum(IV) oxidation state by oxidation of the platinum(II) bpy complexes with chlorine. In addition to the expected platinum(IV) compounds of the type [Pt(bpy)(NH_3_)_2_Cl_2_]^2+^ and [Pt(bpy)Cl_4_], some compounds in which RNH_2_ ligands had been converted to RNHCl species were isolated. This new ligand, which lies along the pathway from NH_3_ to NCl_3_, gave complexes with interesting properties and were reported to “explode violently when heated or even when struck” [190].

Some interesting reports of platinum(II) bpy complexes appeared in 1935 and concerned some anomalous results with the chiral ligand [203,204] H_2_NCHPhCH_2_NH_2_ which were interpreted in terms of an asymmetric platinum atom. The exact origin of these effects is unclear but it seems likely that either the Pfeiffer effect (see later) was operative in the synthesis of complex cations such as [Pt(bpy)(H_2_NCHPhCH_2_NH_2_)]^2+^ or that the authors were observing an early example of diastereoisomerism involving the δ and λ conformations of the chelate ring; the stereochemistry at the H_2_NCHPhCH_2_NH_2_ ligand is known to be either R or S, and the Rδ and Sλ enantiomeric pair are diastereoisomers of the Sδ and Rλ enantiomeric pair.

**Figure 21:**
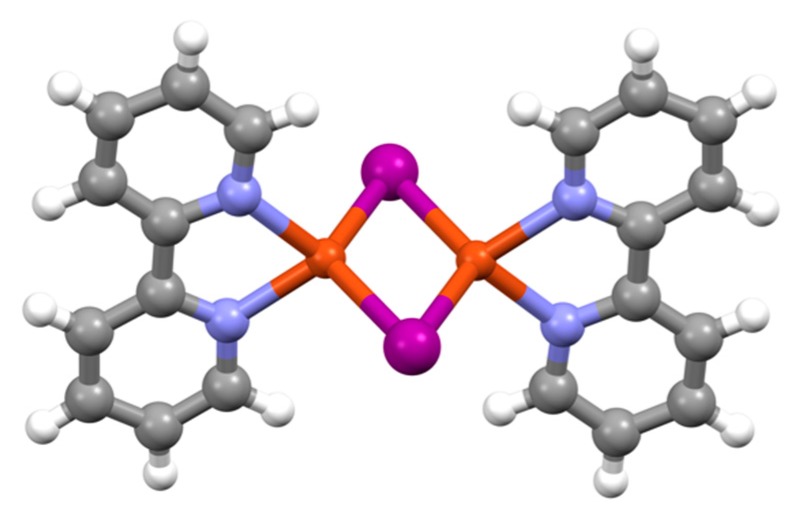
The structure of the dimer [(bpy)(Cu(μ-I)_2_Cu(bpy)] (data taken from reference [207]).

The group 11 elements proved to have an interesting coordination chemistry with bpy ligands and Blau had described copper(II) complexes of bpy [29]. In view of the current interest in copper(I) bpy complexes as photonic materials exhibiting thermally activated delayed fluorescence (TADF), it is appropriate that the first copper(I) species were described as early as 1933. Tartarini reported a series of red-brown complexes with 1:1 and 2:1 bpy:Cu ratios from the reduction of copper(II) complexes or by direct reaction with solid copper(I) compounds [205]. Mann investigated a series of copper(I) compounds and reported that the tetrahedral clusters [(*^n^*Bu_3_E)Cu_4_I_4_] (E = P or As) reacted with bpy to give the mononuclear red complexes [Cu(bpy)(*^n^*Bu_3_E)I] [206]. Heating solutions of [Cu(bpy)(*^n^*Bu_3_P)I] resulted in loss of phosphane and the formation of a compound formulated [(bpy)(Cu(μ-I)_2_Cu(bpy)], identical to one of the materials obtained by Tartarini by the reduction of copper(II) salts with hydrazine and subsequent reaction with bpy and KI. The dinuclear structure has been confirmed by modern crystallographic studies (Figure 21) [207,208].

**Figure 22:**
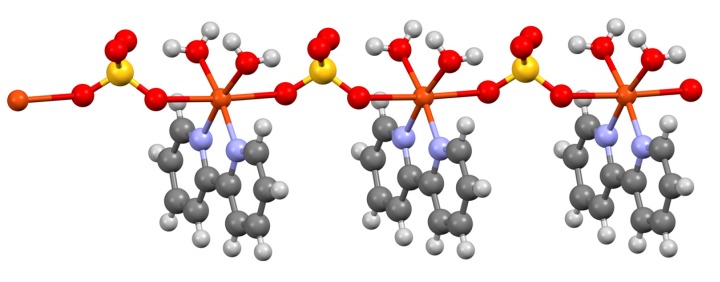
The structure of the one-dimensional coordination polymer [{Cu(bpy)(H_2_O)_2_(μ-SO_4_)}*_n_*] (data taken from reference [211]).

Jaeger investigated the interaction of bpy with copper(II) compounds and described a series of well-characterized species [209]. The reaction of copper(II) propane-1,3-dioate (malonate) with one, two or three equivalents of bpy only gave the 1:1 complex {Cu(bpy)(H_2_O)_2_(C_3_H_2_O_4_)} [116,209]. In contrast, reactions of bpy with aqueous CuSO_4_ gave both pale blue {Cu(bpy)(H_2_O)_2_(SO_4_)} and deep blue [Cu(bpy)_3_](SO_4_).7H_2_O [209]. As might be expected for a labile metal centre exhibiting Jahn-Teller distortions, the structural chemistry of the Cu-bpy-H_2_O-SO_4_ quaternary system is exceptionally complex and diverse. Nevertheless, the compound {Cu(bpy)(H_2_O)_2_(SO_4_)} has been structurally characterized as the one-dimensional coordination polymer [{Cu(bpy)(H_2_O)_2_(μ-SO_4_)}_n_] with bridging bidentate sulfato ligands (Figure 22) [210,211,212,213,214,215,216].

**Figure 23:**
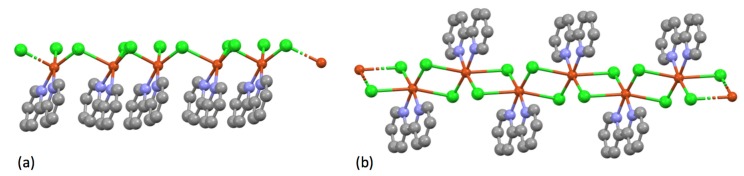
Two polymorphs of {Cu(bpy)Cl_2_}. Although both are one-dimensional coordination polymers, the coordination number of the copper varies. (**a**) [{Cu(bpy)Cl(μ-Cl)}*_n_*] [217], (**b**) [{Cu(bpy)(μ-Cl)_2_}*_n_*] [218].

With CuCl_2_, Jaeger could obtain complexes {Cu(bpy)Cl_2_} (green), {Cu(bpy)_2_(H_2_O)_2_Cl_2_} (blue) and violet [Cu(bpy)_3_]Cl_2_.7H_2_O [209]. Despite the simple stoichiometry, {Cu(bpy)Cl_2_} exhibits an unexpected structural complexity and a number of compounds have been characterized. The simplest is a one-dimensional coordination polymer [{Cu(bpy)Cl(μ-Cl)}*_n_*] with five-coordinate copper centres, each coordinated to one bidentate bpy ligand, one terminal chlorine (2.259 Å) and two bridging chlorines (2.291 and 2.674 Å) (Figure 23a) [217]. The remaining compounds are also one-dimensional coordination polymers [{Cu(bpy)(μ-Cl)_2_}*_n_*] with six-coordinate copper centres, each coordinated to one bidentate bpy ligand and four bridging chlorines exhibiting two short Cu-Cl bonds (2.2 to 2.5 Å) and two longer Cu…Cl (2.9 to 3.2 Å) interactions (Figure 23b) [218,219,220,221,222]. In a second publication, Jaeger described the formation of compounds formulated {Cu(bpy)(H_2_O)_3_(NO_3_)_2_}, {Cu(bpy)_2_(H_2_O)(NO_3_)_2_}, {Cu(bpy)_3_](NO_3_)_2_.6H_2_O} [223]. The compound {Cu(bpy)(H_2_O)_3_(NO_3_)_2_} has been structurally characterized and shown to be the mononuclear species [Cu(bpy)(H_2_O)_3_(ONO_2_)](NO_3_) [224,225,226,227]; although {Cu(bpy)_2_(H_2_O)(NO_3_)_2_} has not been structurally characterized, the related compounds [Cu(bpy)_2_(ONO_2_)](NO_3_).0.5H_2_O [228] [Cu(bpy)_2_(ONO_2_)](NO_3_) [229,230,231,232,233,234] [Cu(bpy)_2_(ONO_2_)](NO_3_).H_2_O [235,236,237] all contain six-coordinate copper centres with two bidentate bpy ligands and a chelating bidentate nitrato ligand.

**Figure 24:**
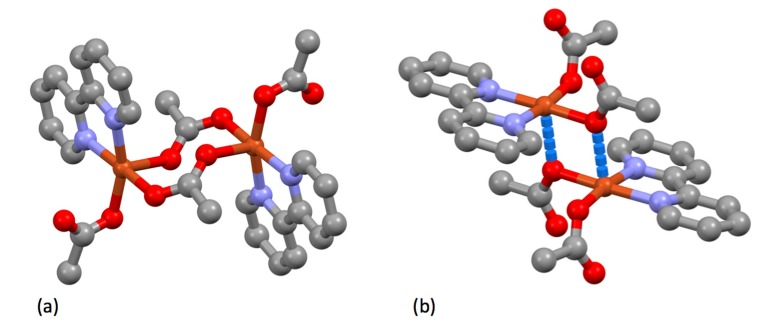
(**a**) The dimeric structure of [(AcO)(bpy)Cu(μ-OAc)_2_Cu(AcO)(bpy)].2H_2_O [240] and (**b**) the dimer present in anhydrous [Cu(bpy)(OAc)_2_] [241].

In addition to the compounds described above, Jaeger also reported the formation and described the crystal morphology of {Cu(bpy)(OAc)_2_.(H_2_O)_5_} [116] and a further series of compounds including [Cu(bpy)_3_](ClO_4_)_2_ and mixed ligand complexes with bpy and the amino acids l-valine, isoleucine and l-alanine [238]. As is always the case, some of these simple compounds proved to be significantly more complex and diverse than expected. For example, of [Cu(bpy)(OAc)_2_].5H_2_O is mononuclear and best described as a square-planar copper(II) centre with two short (1.941 Å) contacts; however, the remaining acetate oxygen atoms then form pseudo-axial interactions (∠Oax-Cu-Oax 126.25°) with longer Cu...O contacts of 2.61 Å [239]. In contrast, the monohydrate [Cu(bpy)(OAc)_2_].H_2_O is formulated as [(AcO)(bpy)Cu(μ-OAc)_2_Cu(AcO)(bpy)].2H_2_O (Figure 24a) [240] whereas the anhydrous [Cu(bpy)(OAc)_2_] is a dimer of two square-planar {Cu(bpy)_2_(OAc)_2_} moieties held together by short Cu...O interactions of 2.384 Å (Figure 24b) [241].

**Figure 25:**
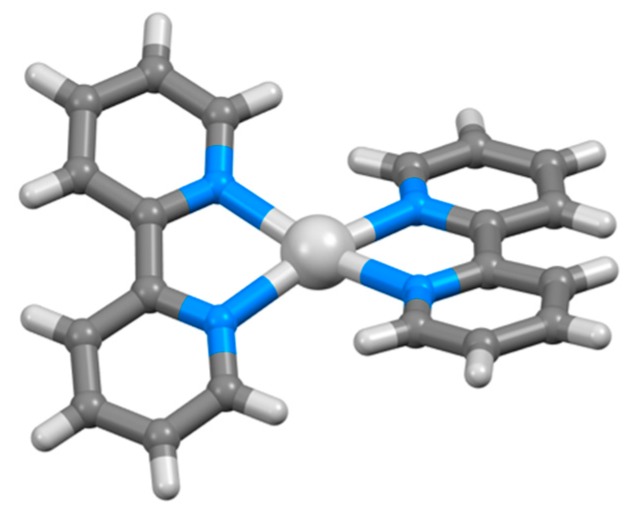
The solid-state structure of [Ag(bpy)_2_]^2+^ cation in [Ag(bipy)_2_](ClO_4_)_2_ [245].

One of the earliest studies on bpy complexes by Morgan concerned silver(II) complexes, which were prepared by the oxidation of [Ag(bpy)_2_]^+^ with persulfate [242]. The use of bpy to stabilize an "unusual" oxidation state in one of the pioneering studies of its coordination chemistry seems appropriate and a prophecy of the vital role this ligand was to play in the development and understanding of coordination chemistry. The complex [Ag(bpy)_2_][NO_3_] was obtained directly from bpy and AgNO_3_ and is diamagnetic [243]. After persulfate oxidation, silver(II) complexes with both a 2:1 and 3:1 bpy:Ag ratio were isolated. The 2:1 compounds were also prepared by electrolysis of solutions containing AgNO_3_ in the presence of bpy and in this way, [Ag(bpy)_2_](ClO_4_)_2_ and [Ag(bpy)_2_](NO_3_)_2_ were obtained [244]. Hein described the preparation of the silver(I) compound [Ag(bpy)_2_](NO_3_) and its oxidation by K_2_S_2_O_8_ to the silver(II) compound [Ag(bpy)_2_](S_2_O_8_) [85]. The [Ag(bpy)_2_]^2+^ cation is well-established in compounds such as [Ag(bpy)_2_](ClO_4_)_2_ [245] and [Ag(bpy)_2_](NO_3_)_2_⋅H_2_O [246]; these complexes are not planar but have angles between the least squares planes through the near-planar bpy ligands of 35.17° and 32.25° respectively (Figure 25). In [Ag(bpy)_2_](NO_3_)_2_⋅H_2_O, there are also longer Ag...O interactions to oxygen atoms of nitrate anions at > 2.8 Å. The nature of the claimed [Ag(bpy)_3_]^2+^ species is less clear; although magnetic [243,247], electron paramagnetic resonance [248] and electronic spectra [249] have been reported, the existence of these compounds has been cast in doubt [250], and to date only the [Ag(bpy)_2_]^2+^ compounds have been established unequivocally. Magnetic data were also reported for compounds [Ag(bpy)_2_](S_2_O_8_) and {Ag_2_(bpy)_5_(S_2_O_8_)_2_} [243]. Hiebert described the complex (Ag(bpy)[Co(CO)_4_] [251].

**Figure 26:**
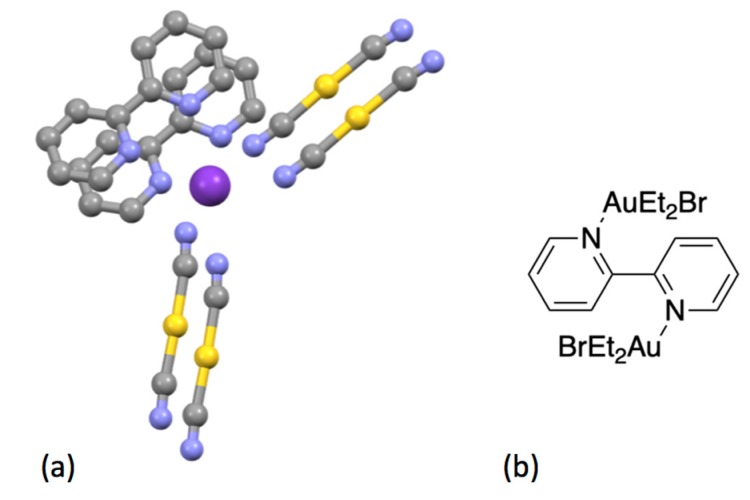
(**a**) The compound initially formulated K[Au(bpy)(CN)_2_] was subsequently shown to have no bpy-Au bonding (data taken from reference 253. Colour code: K purple; Au, yellow. (**b**) The proposed structure of [Et_2_AuBr(μ-bpy)AuBrEt_2_] [255].

The first gold bpy complex to be claimed was {KAu(bpy)(CN)_2_}, which was obtained from the reaction of K[Au(CN)_2_] with bpy and reported to contain a square-planar [Au(bpy)(CN)_2_]^–^ ion, on the basis of X-ray crystallographic data showing a very short (3.74 Å) *b*-axis [252]. Some 40 years later, the crystal structure of the compound was determined and it was shown to contain linear [Au(CN)_2_]^–^ anions; the bpy was not coordinated to the gold but adopts a cisoid conformation and interacts with the potassium cations at 2.778 and 3.080 Å (Figure 26a) [253,254]. In view of the current interest in organogold compounds in organic synthesis, it is appropriate that one of the first gold complexes containing a bpy ligand should be an organometallic compound [255]. Attempts to form the five-coordinate gold complex [Et_2_Au(bpy)Br] from the reaction of [Et_2_Au(μ-Cl)_2_AuEt_2_] with bpy, gave only [Et_2_AuBr(μ-bpy)AuBrEt_2_], the first example of a compound containing a ditopic bpy ligand acting as a bridging bidentate species presenting one pyridine metal-binding domain to each of two {Et_2_AuBr} moieties (Figure 26b).

**Figure 27:**
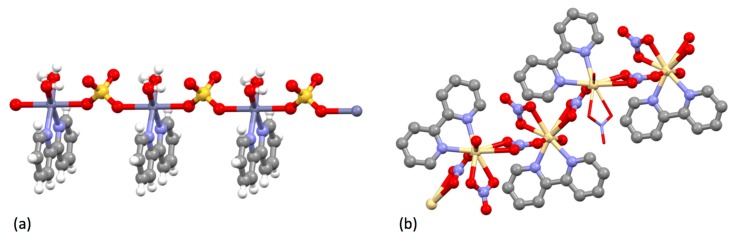
The one dimensional coordination polymers (**a**) [{Zn(bpy)(H_2_O)_2_(μ-SO_4_)}_n_] (data taken from reference [260]) and (**b**) [{Cd(bpy)(H_2_O)(O_2_NO)(μ-NO_3_)}_n_] [269].

The group 12 elements have a rich and varied bpy coordination chemistry. Blau had described zinc(II) complexes, but it was left to Paul Pfeiffer to report in detail the complexes containing the [Zn(bpy)_3_]^2+ ^ and [Cd(bpy)_3_]^2+^ ions [256,257]. These compounds were critical in the identification and understanding of the Pfeiffer effect in which an enantiopure or enantioenriched compound enhances the resolution of a racemic mixture of a second compound. The effect originates in supramolecular interactions between the chiral species and the second component (in this case, the enantiomeric Λ and Δ [M(bpy)_3_]^2+^ cations) to generate diastereoisomeric contact pairs with different free energies. The zinc(II) and cadmium(II) complexes are labile, and this in turn leads to an enrichment of the diastereoisomeric contact pair with the lowest energy when they interact with an enantiopure species. For example, interacting with a second species of R chirality, the diastereoisomeric contact pairs {ΛR} and {ΔR} have different energies, and as the Λ and Δ are in dynamic equilibrium, the solution becomes enriched in the lower energy {ΛR} or {ΔR} component. As the chirality of the additional species is fixed, the lowest energy diastereoisomer is attained through an enrichment of either the Λ or Δ [M(bpy)_3_]^2+^ cation. Pfeiffer demonstrated the enhancement with sub-stoichiometric amounts of camphorsulfonic acid and bromocamphorsulfonic acid. The lability of the zinc and cadmium complexes was further illustrated by Jaeger, who obtained not only [M(bpy)_3_]^2+^ complexes, but also species with 1:1 and 2:1 bpy:M ratios [258]. Although the reaction of ZnSO_4_ with two equivalents of bpy gave equimolar amounts of [Zn(bpy)_3_]^2+^ and {Zn(bpy)(H_2_O)_2_(SO_4_)}, with Cd(NO_3_)_2_, the complex {Cd(bpy)_2_(NO_3_)_2_(H_2_O)_0.5_} was obtained. The compound {Zn(bpy)(H_2_O)_2_(SO_4_)} has subsequently been shown to be a one-dimensional coordination polymer in which planar {Zn(bpy)(H_2_O)_2_} moieties are linked by bridging bidentate sulfato anions (Figure 27) [215,259,260,261,262,263,264] whereas {Cd(bpy)_2_(NO_3_)_2_(H_2_O)_0.5_} is a mixed crystal containing a stoichiometric mixture of [Cd(bpy)_2_(NO_3_)_2_] and [Cd(bpy)_2_(NO_3_)(H_2_O)](NO_3_) [265,266,267]. In other studies, Jaeger described the synthesis and crystal morphology of various [M(bpy)_3_]^2+^ salts (M = Cd, Zn) and of compounds including {Zn(bpy)(H_2_O)*_n_*(NO_3_)_2_}, {Zn(bpy)(H_2_O)*_n_*Cl_2_}, {Cd(bpy)(H_2_O)*_n_*(NO_3_)_2_}, {Cd(bpy)(H_2_O)*_n_*(SO_4_)}, and {Cd(bpy)_2_(H_2_O)Cl_2_} [116,268]. The compound {Cd(bpy)(H_2_O)*_n_*(NO_3_)_2_} has been structurally characterized and shown to be the one-dimensional coordination polymer [{Cd(bpy)(H_2_O)(O_2_NO)(μ-NO_3_)}*_n_*] in which the cadmium has a bicapped trigonal-antiprismatic coordination geometry comprising a bidentate bpy, a bidentate nitrato ligand, a water and two bridging nitrato ligands (one bidentate, the other monodentate) (Figure 27b) [269].

One of the earliest compounds of bpy with a group 12 element to be described was [Hg(bpy)I_2_], which Morgan identified as an insoluble species, which could be used for the gravimetric determination of bpy [242]. Like many of these apparently simple species, the compound has been subsequently structurally characterized and shown to possess a more complex structure with a very distorted four-coordinate mercury centre [270]. Pfeiffer reported the salt (bpyH)[HgCl_3_], which presumably has no bpy-Hg interactions [271].

#### 5.2.3. Complexes of Elements in Groups 13 to 18

Pfeiffer showed that the reaction of lead nitrate with bpy in aqueous MeOH followed by treatment with NaClO_4_ gave {Pb(bpy)_2_(ClO_4_)_2_} as a colourless crystalline product [271]. No complexes with a greater bpy to lead ratio could be isolated.

### 5.3. Analytical Applications

Although colorimetric analysis had a distinguished pedigree, it was not widely utilized until the beginning of the 20th century CE. Particularly influential was the publication of the first book written in English on the method in 1921 [272] after which a steady increase in the number of publications followed. In the course of the 1930s, commercial colorimeters became more readily available and reagents for colorimetric analytical determination of specific metals were developed. The colorimetric methods revolutionalized the analysis of biological materials and allowed the determination of molecular and ionic species in a vast array of physiologically active species or metabolites [273]. The quantitative analysis of iron was of especial interest and the intense red colour of the [Fe(bpy)_3_]^2+^ cation (ε = 8650 M^−1^ cm^−1^, λ_max_ = 522 nm) attracted early attention. Hill [274] and others [106,119,275,276,277,278,279,280,281,282,283,284,285] developed colorimetric methods for the determination of iron as [Fe(bpy)_3_]^2+^ in biological materials. McFarlane noted the similarity of the absorption spectra of aqueous cobalt(II) nitrate solutions and [Fe(bpy)_3_]^2+^ and proposed the former as a colorimetric standard for calibration [279]. This colorimetric method rapidly became the method of choice for the analysis of iron in a wide range of materials and the technique was applied, inter alia, to the determination of the iron content of hematin and cytochrome c [286], water [287,288,289,290], milk [291], beer [292,293], other foodstuffs [294,295,296,297,298,299,300,301], soil and minerals [302,303,304,305,306], teeth [307,308], blood [285,309,310,311,312,313,314] and other biological materials [315,316,317,318,319] and homeopathic formulations [320]. It was recognized that the methods using bpy for the determination of iron gave a measure of the "available" iron, and any Fe that was strongly bound in heme proteins or ferritin would not necessarily be included in the total iron [321,322]. In view of the emerging recognition that bpy formed complexes with most metals investigated, the effects of other metal ions on the accuracy of the determination of iron was studied [284,323,324]. Protocols were developed for the orthogonal determination of iron and copper in biological samples using bpy and carbamate, respectively [325].

An interesting application of bpy was reported by Koenig, who developed a method for the determination of aluminium as Al_2_O_3_ in which interference by iron(III) was minimized by a protocol in which the iron was reduced by hydroxylamine and coordinated to bpy before the aluminium oxide was precipitated. The thermodynamic and kinetic stability of [Fe(bpy)_3_]^2+^ is sufficiently high that Fe_2_O_3_ was not precipitated under these conditions [326].

The red colour is specific to the iron(II) complex of bpy, but methods for the analysis of iron(III) or materials containing iron in multiple oxidation states were developed involving reaction with bpy (or phen) in the presence of a reducing agent such as ascorbic acid [327], phenols [328] or sulfur compounds [328]. Various modifications of the analytical protocols were described for turbid, highly coloured or fresh biological samples [280].

The characteristic coloured complexes of bpy with other metals were also used for their analysis. In the presence of the reducing agent tin(II) chloride, molybdenum compounds gave a characteristic deep red-violet coloration allowing the detection of Mo down to micromolar concentrations [98,99]. Salts of [Fe(bpy)_3_]^2+^ with inorganic anions are typically insoluble making them of use in the microchemical analysis of these species [278] and was utilized in an early method for the qualitatitive detection of Ta and Nb by the precipitation of characteristic crystalline tantalate or niobate salts [329].

In addition to the direct determination of iron by bpy, the complexes [Fe(bpy)_3_]^2+/3+^ have found application as redox indicators. The standard redox potential, E° of this couple is ≈ +1.08 V in aqueous solution and the reduction of blue [Fe(bpy)_3_]^3+^ to red [Fe(bpy)_3_]^2+^ is accompanied by a dramatic visual change. An early review of iron coordination chemistry dating from 1928 appears to be the first recognition of Blau’s observation of the redox couple [330]. With a subsequent colorimetric determination of iron(II) as [Fe(bpy)_3_]^2+^, methods for the determination of vitamin C (ascorbic acid) [331], vitamin E (tocopherol) [332,333,334,335,336,337] were developed based upon the quantitative reduction of Fe^3+^ by the analytes. Most of these methods utilize mixtures of bpy and iron(III) compounds, which in the presence of reducing agents that can reduce the iron(III) to iron(II) develop the characteristic red [Fe(bpy)_3_]^2+^ colour which can be quantified colorimetrically.

### 5.4. Biological Studies

In studies on the activation of arginase by iron complexes, rats with iron-deficient livers were obtained after a diet containing bpy [338]. It had previously been established that feeding rats with bpy resulted in decreased absorption of iron and consequent and inhibition of hemoglobin formation [339]. In parallel, it was shown that [Fe(bpy)_3_]^2+^ itself could activate arginase [340]. However, studies of the absorption, metabolism and physiological impact of [Fe(bpy)_3_](SO_4_) showed that modest blood levels could cause convulsions, inhibit respiration and have occular effects [341,342]. Another early study showed that [Fe(bpy)_3_](SO_4_), together with other iron compounds, deactivated calf spleen proteases [343]. Although catalase is inhibited by simple iron(II) salts, neither bpy nor [Fe(bpy)_3_]^2+^ salts affect its activity [344]. Neither pepsin nor the cathepsins are inhibited by bpy [345]. Although the biological effects of [Fe(bpy)_3_]^2+^ were beginning to be documented, the stability of the complex led Eicholtz to classify it with the porphyrins and the hexacyanoferrates as a type of compound in which the iron is not biologically available [346].

### 5.5. Physicochemical Properties

The 1930s were characterized by the development of new spectroscopic and physicochemical methods as well as the increasing availability and utilization of instrumentation for routine measurements.

Jaeger described the crystalline form of bpy as early as 1934 [86] and the first Raman spectrum of bpy was reported by Bonino in 1934 [347]. In two remarkable early papers, Yamasaki reported the electronic absorption spectra of the complexes [M(bpy)_3_]Cl_2_ (M = Cu, Co, Zn, Ni, Fe), [Mn(bpy)_2_Cl_2_], [Co(bpy)_2_Cl_2_]Cl, [Co(bpy)_2_(CO_3_)]Cl and [Co(bpy)_3_]Cl_3_ [348,349]. Every modern chemist should read these papers to appreciate how non-trivial such measurements were in the 1930’s.

### 5.6. Other Applications

In a very early example of what we would now call organocatalysis, the effects of various additives on the autoxidation of linoleic acid and related compounds was studied, and bpy shown to be rather effective [350].

## 6. Conclusions

We have followed the story of the ligand 2,2′-bipyridine from its birth to the celebration of its fiftieth birthday in 1939. In these fifty years, we have seen coordination chemistry develop from the first days of the Werner model to a fully established discipline. Along the way, the fundamental aspects of coordination chemistry were debated and chelating ligands such as bpy played a crucial role in the development of our modern understanding. At the same time, techniques for investigating the structures of molecules in ever more intimate detail were developing, giving us an insight into the nature of matter. One feature that emerges from the coordination chemistry studies, is that many “simple” compounds had structural complexities that were not suspected when first reported.

The story of bpy is not over. In its second century of life, bpy continues to be one of the metal-binding domains of choice for introduction into supramolecular and nanoscale materials. I am certain that bpy ligands will continue to serve us, occasionally confuse us and always edify us.

## Figures and Tables

**Table 1:** pKa values for protonated 2,2′-bipyridine, 1,10-phenanthroline and pyridine in aqueous solution. Data for bpy and phen taken from reference [44].

Compound	pKa
(bpyH_2)_^2+^	0.05
(bpyH)^+^	4.4
(phenH_2_)^2+^	−0.2
(phenH)^+^	4.9
(pyH)^+^	5.25

## References

[1] Kaes C., Katz A., Hosseini M.W. (2000). Bipyridine: The most widely used ligand. A review of molecules comprising at least two 2,2′-bipyridine units. Chem. Rev..

[2] Summers L.A. (1984). The bipyridines. Adv. Heterocycl. Chem..

[3] Summers L.A. (1978). The phenanthrolines. Adv. Heterocycl. Chem..

[4] Constable E.C. (1989). Homoleptic complexes of 2,2′-bipyridine. Adv. Inorg. Chem..

[5] Accorsi G., Listorti A., Yoosaf K., Armaroli N. (2009). 1,10-Phenanthrolines: Versatile building blocks for luminescent molecules, materials and metal complexes. Chem. Soc. Rev..

[6] Bencini A., Lippolis V. (2010). 1,10-Phenanthroline: A versatile building block for the construction of ligands for various purposes. Coord. Chem. Rev..

[7] Lindoy L.F., Livingstone S.E. (1967). Complexes of iron(II),cobalt(II) and nickel(II) with α-diimines and related bidentate ligands. Coord. Chem. Rev..

[8] McWhinnie W.R., Miller J.D. (1970). The chemistry of complexes containing 2,2′-bipyridyl, 1, 10-phenanthroline, or 2,2′,6′,2”-terpyridyl as ligands. Adv. Inorg. Chem. Radiochem..

[9] Brandt W.W., Dwyer F.P., Gyarfas E.D. (1954). Chelate complexes of 1,10-phenanthroline and related compounds. Chem. Rev..

[10] Constable E.C. (1986). The Coordination Chemistry of 2,2′:6′,2″-Terpyridine and Higher Oligopyridines. Adv. Inorg. Chem..

[11] Fallahpour R.-A. (2006). The Higher Oligopyridines and their Metal Complexes. Curr. Org. Synth..

[12] Constable E.C. (1994). Higher Oligopyridines as a Structural Motif in Metallosupramolecular Chemistry. Prog. Inorg. Chem..

[13] Schubert U.S., Winter A., Newkome G.R. (2011). Terpyridine-Based Materials: For Catalytic, Optoelectronic and Life Science Applications.

[14] Constable E.C. (2007). 2,2′:6′,2″-Terpyridines: From chemical obscurity to common supramolecular motifs. Chem. Soc. Rev..

[15] Schubert U.S., Hofmeier H., Newkome G.R. (2006). Modern Terpyridine Chemistry.

[16] Blau F. (1888). Die Destillation pyridinmonocarbonsaurer Salze. Ber. Dtsch. Chem. Ges..

[17] Weidel H. (1879). Studien über Verbindungen aus dem animalischen Theer. Ber. Dtsch. Chem. Ges..

[18] Anderson T. (1870). Ueber die Producte der trockenen Destillation thierischer Materien. Ann. Chem. Pharm..

[19] Anderson T. (1869). On the products of the destructive distillation of animal substances. J. Chem. Soc..

[20] Weidel H., Russo M. (1882). Studien über das Pyridin. Monatsh. Chem..

[21] Emmert B. (1917). Über Verbindungen des Pyridins mit den Alkalimetallen. III. Mitteilung. Ber. Dtsch. Chem. Ges..

[22] Smith C.R. (1924). Dipyridyls from pyridine. J. Am. Chem. Soc..

[23] Hartley W.N. (1885). Researches on the relation between the molecular structure of carbon compounds and their absorption spectra. (Part VII). J. Chem. Soc. Trans..

[24] Heuser A., Stoehr C. (1890). 4. Ueber methylirte Dipyridyle; I. Abhandlung. J. Prakt. Chem..

[25] Heuser A., Stoehr C. (1891). Ueber methylirte Dipyridyle; II. Abhandlung. J. Prakt. Chem..

[26] Stoehr C., Wagner M. (1893). Ueber methylirte Dipyridyle. J. Prakt. Chem..

[27] Blau F. (1889). Über die trockene Destillation von pyridincarbonsauren Salzen. Monatsh. Chem..

[28] Blau F. (1889). Neuerungen beim gebräuchlichen Verbrennungsverfahren. Monatsh. Chem..

[29] Blau F. (1898). Über neue organische Metallverbindungen. Monatsh. Chem..

[30] Werner A. (1893). Beitrag zur Konstitution anorganische Verbindungen. Z. Anorg. Chem..

[31] Tobies R. (2012). Iris Runge: A Life at the Crossroads of Mathematics, Science, and Industry.

[32] Tobies R. (2010). Morgen möchte ich wieder 100 herrliche Sachen ausrechnen. Iris Runge bei Osram und Telefunken.

[33] Pirani M. (1930). Fritz Blau. Naturwissenschaften.

[34] Favre H.A., Powell W.H. (2013). Nomenclature of Organic Chemistry—IUPAC Recommendations and Preferred Names 2013.

[35] Connelly N.G., Damhus T., Hartshorn R.M., Hutton A.T. (2005). Nomenclature of Inorganic Chemistry: IUPAC Recommendations; Issued by the Division of Chemical Nomenclature and Structure Representation in collaboration with the Division of Inorganic Chemistry.

[36] Favre H.A., Powell W.H. (2013). Nomenclature of Organic Chemistry—IUPAC Recommendations and Preferred Names 2013.

[37] Groom C.R., Bruno I.J., Lightfoot M.P., Ward S.C. (2016). The Cambridge Structural Database. Acta Crystallogr. Sect. B Struct. Sci. Cryst. Eng. Mater..

[38] Merritt jr L.L., Schroeder E.D. (1956). The crystal structure of 2,2′-bipyridine. Acta Crystallogr..

[39] Nakatsu K., Yoshioka H., Matsui M., Koda S., Ooi S. (1972). Structural studies on 2,2′-bipyridinium and 1,10-phenanthrolinium cations. Acta Crystallogr. Sect. A Cryst. Phys. Diffr. Theor. Gen. Crystallogr..

[40] Cagle F.W. (1948). A preliminary examination of the crystal structure of 2,2′-bipyridyl and its relation to biphenyl. Acta Crystallogr..

[41] Chisholm M.H., Huffman J.C., Rothwell I.P., Bradley P.G., Kress N., Woodruff W.H. (1981). Bis(2,2′-bipyridyl)diisopropoxymolybdenum(II). Structural and spectroscopic evidence for molybdenum-to-bipyridyl π* bonding. J. Am. Chem. Soc..

[42] Kuhn F.E., Groarke M., Bencze E., Herdtweck E., Prazeres A., Santos A.M., Calhorda M.J., Romao C.C., Goncalves I.S., Lopes A.D. (2002). Octahedral bipyridine and bipyrimidine dioxomolybdenum(VI) complexes: Characterization, application in catalytic epoxidation, and density functional mechanistic study. Chem. Eur. J..

[43] Alkorta I., Elguero J., Roussel C. (2011). A theoretical study of the conformation, basicity and NMR properties of 2,2′-, 3,3′- and 4,4′-bipyridines and their conjugated acids. Comput. Theor. Chem..

[44] Krishnan C.V., Creutz C., Schwarz H.A., Sutin N. (1983). Reduction potentials for 2,2′-bipyridine and 1,10-phenanthroline couples in aqueous solutions. J. Am. Chem. Soc..

[45] Constable E.C., Housecroft C.E. (2017). More Hydra than Janus—Non-classical coordination modes in complexes of oligopyridine ligands. Coord. Chem. Rev..

[46] Purvis J.E. (1913). The absorption spectra of various derivatives of pyridine, piperidine, and piperazine in solution and as vapours. J. Chem. Soc. Trans..

[47] IUPAC Gold Book. https://goldbook.iupac.org/terms/view/A00511.

[48] Mascarelli L. (1928). Diphenyl and its derivatives (and dinaphthyls). Cleavage into optical antipodes of compounds which do not contain asymmetric atoms. II. Gazz. Chim. Ital..

[49] Summers L.A. (1980). The Bipyridinium Herbicides.

[50] Richardson C.H., Smith C.R. (1923). Studies on Contact Insecticides.

[51] Richardson C.H., Smith C.R. (1923). Toxicity of dipyridyls and certain other organic compounds as contact insecticides. J. Agric. Res..

[52] Smith C.R., Richardson C.H., Shepard H.H. (1930). Neonicotine and certain other derivatives of the dipyridyls as insecticides. J. Econ. Entomol..

[53] Smith C.R. (1931). Neonicotine and isomeric pyridylpiperidines. J. Am. Chem. Soc..

[54] Emmert B. (1914). Über die Verbindungen des Pyridins und Picolins mit metallischem Natrium. Ber. Dtsch. Chem. Ges..

[55] Emmert B. (1916). Über Verbindungen des Pyridins mit den Alkalimetallen. Ber. Dtsch. Chem. Ges..

[56] Emmert B., Buchert R. (1921). Über Verbindungen des Pyridins mit den Alkalimetallen (IV). Ber. dtsch. Chem. Ges. A/B.

[57] Weitz E., Nelken A., Ludwig R. (1921). Das Benzylpyridinium. Justus Liebigs Ann. Chem..

[58] Hofmann A.W. (1881). Zur Geschichte der Pyridinbasen. Ber. Dtsch. Chem. Ges..

[59] Emmert B. (1919). Über die Konstitution der von A. W. Hofmann aufgefundenen Dialkyl-tetrahydro-dipyridyle. Ber. Dtsch. Chem. Ges. A/B.

[60] Weitz E., Ludwig R. (1922). Über freie Ammonium Radikale, III. Mitteilung: Die Existenz des N-Benzyl-pyridiniums. Ber. Dtsch. Chem. Ges. A/B.

[61] Weitz E., König T., von Wistinghausen L. (1924). Über freie Ammonium-Radikale, V.: Vergleich von N,N′-Dibenzyl- und N,N′-Diphenyl-γ,γ′-dipyridinium. Ber. Dtsch. Chem. Ges. A/B.

[62] Weitz E., König T. (1922). Über freie Ammoniumradikale, IV. Mitteilung: Weitere Untersuchungen über das NN′-Dibenzyl-γ,γ′-dipyridinium und seine Homologen, sowie die sog. NN′-disubstituierten Tetrahydro-γ,γ′-dipyridyle. Ber. Dtsch. Chem. Ges. A/B.

[63] Emmert B., Varenkamp O. (1922). Über N,N′-Dialkyl-[tetrahydro-γ,γ′-dipyridyle]. Ber. Dtsch. Chem. Ges. A/B.

[64] Emmert B., Jungck G., Häffner H. (1924). Über chinhydron-artige Verbindungen des Dihydro-γ,γ-dipyridyls. Ber. Dtsch. Chem. Ges. A/B.

[65] Mumm O., Ludwig H. (1926). Zur Kenntnis der N,N′-Dialkyl-[tetrahydro-dipyridyle]. Ber. Dtsch. Chem. Ges. A/B.

[66] Mumm O., Roder O., Ludwig H. (1924). Zur Kenntnis der N,N′-Dialkyl-[tetrahydro-dipyridyle]. Ber. Dtsch. Chem. Ges. A/B.

[67] Emmert B., Werb O. (1922). Über N,N′-Dimethyl-[tetrahydro-γ,γ′-dikollidyl]. Ber. Dtsch. Chem. Ges. A/B.

[68] Emmert B., Varenkamp O. (1923). Über chinhydronartige Verbindungen der N,N′-Dialkyl-[dihydro-γ,γ′-dipyridyle]. Ber. Dtsch. Chem. Ges. A/B.

[69] Dimroth O., Heene R. (1921). Reduktion von Pyridin mit Zinkstaub und Essigsäure-anhydrid. Ber. Dtsch. Chem. Ges. A/B.

[70] Dimroth O., Frister F. (1922). Reduktion von Pyridin mit Zinkstaub und Essigsäure-anhydrid (2. Mitteilung). Ber. Dtsch. Chem. Ges. A/B.

[71] Fanta P.E. (1974). The Ullmann Synthesis of Biaryls. Synthesis.

[72] Wibaut J.P., Overhoff J. (1928). Über eine neue Darstellungsmethode von 2,2′-Dipyridyl. Recl. Trav. Chim. Pays Bas.

[73] Meyer H., Hofmann-Meyer A. (1921). Über Pyrokondensationen in der Pyridinreihe. J. Prakt. Chem..

[74] Wibaut J.P., van de Lande L.M.F. (1929). Sur la Formation de L’aminopyridine par L’action du gaz Ammoniac sur la Pyridine en Présence de Catalyseurs. Recl. Trav. Chim. Pays-Bas.

[75] Wibaut J.P., Dingemanse E. (1926). Über eine Pyrogene Umlagerung von n-Methyl-(α-Pyridyl)-α-Pyrrol. Recl. Trav. Chim. Pays-Bas.

[76] Sabatier P., Fernandez A. (1927). Déshydrogénations et hydrogénations catalysées par des oxydes métalliques. C. R. Hebd. Seances Acad. Sci..

[77] Hein F., Retter W. (1928). Eine neue Methode zur Darstellung von α,α′-Dipyridyl. Ber. Dtsch. Chem. Ges. A/B.

[78] Werner A. (1912). Über Spiegelbild-Isomerie bei Eisenverbindungen. Ber. Dtsch. Chem. Ges..

[79] Freudlich H., Birstein V. (1926). Über einige Eigenschaften der Blauschen Komplexsalze. Kolloidchem. Beih..

[80] Morgan G.T., Drew H.D.K. (1920). Researches on residual affinity and co-ordination. Part II. Acetylacetones of selenium and tellurium. J. Chem. Soc. Trans..

[81] Lowry T.M. (1923). Stability of co-ordination compounds. J. Soc. Chem. Ind. London Rev. Sect..

[82] Smith J.D.M. (1923). Chelate co-ordination. J. Soc. Chem. Ind. Lond. Rev. Sect..

[83] Lowry T.M. (1923). Stability of co-ordination compounds. J. Soc. Chem. Ind. London Rev. Sect..

[84] Biltz W. (1928). Über Molekular- und Atomvolumina. XVII. Zur Raumchemie und Magnetochemie fester Cyanide. Z. Anorg. Allg. Chem..

[85] Hein F., Schwedler H. (1935). Zur α, α′-Dipyridyl-Synthese aus Pyridin und Eisen(III)-chlorid, sowie über einige Dipyridyl-Komplexsalze. Ber. Dtsch. Chem. Ges. B.

[86] Jaeger F.M., van Dijk J.A. (1934). Complex salts with α,α′-dipyridyl. Complex salts of bivalent nickel. Proc. K. Ned. Akad. Wet..

[87] Morgan G.T., Burstall F.H. (1932). Dehydrogenation of pyridine by anhydrous ferric chloride. J. Chem. Soc..

[88] Morgan G.T., Burstall F.H. (1933). Dehydrogenation of pyridine by anhydrous metallic chlorides. J. Indian Chem. Soc. Prafulla Chandra Ray Commemoration Vol..

[89] Willink H.D.T., Wibaut J.P. (1935). Preparation of 2,2′-bipyridyl and some of its derivatives. Recl. Trav. Chim. Pays-Bas.

[90] Thate H. (1931). The hydrogenation of pyridine with hydrogen under pressure by the Bergius process. Recl. Trav. Chim. Pays-Bas.

[91] Burstall F.H. (1938). Researches on the Polypyridyls. J. Chem. Soc..

[92] Wibaut J.P., Willink H.D.T. (1931). Eine Darstellungsmethode von 22″-Dipyridyl durch Katalytische Dehydrierung von Pyridin unter Druck. Recl. Trav. Chim. Pays-Bas.

[93] Wibaut J.P., Van De Lande L.M.F. (1931). Über die Einwirkung von Ammoniakgas auf Pyridin und 2-Methylpyridin in Gegenwart von Dehydrogenations-Katalysatoren. (Zweite Mitteilung). Recl. Trav. Chim. Pays-Bas.

[94] Kabachnik M.I. (1935). The mechanism of the reaction of pyridine and its derivatives with the alkali metal amides. Bull. Acad. Sci. U. R. S. S. Classe Sci. Math. Nat..

[95] Smith C.R. (1930). Skraup’s reaction applied to the phenylenediamines. Preparation of the phenanthrolines and related bipyridyls. J. Am. Chem. Soc..

[96] Barbieri G.A., Tettamanzi A. (1932). Compounds of bivalent chromium. ATTI Accad. Naz. Lincei Cl. Sci. Fis. Mat. Nat. Rend..

[97] Balthis J.H., Bailar J.C. (1936). Some chromous and chromic ammines. J. Am. Chem. Soc..

[98] Komarovskii A.S., Poluektov N.S. (1937). Color reaction of molybdenum with α,α‘-bipyridyl. Zh. Prikl. Khim..

[99] Komarovskii A.S., Poluektov N.S. (1937). Über eine Farbreaktion auf Molybdän mit α-α′-Dipyridyl. Microchim. Acta.

[100] Hieber W., Romberg E. (1935). Über Metallcarbonyle. XXII. Derivate des Wolframhexacarbonyls. Z. Anorg. Allg. Chem..

[101] Ye Q., Wu Q., Zhao H., Song Y.-M., Xue X., Xiong R.-G., Pang S.-M., Lee G.-H. (2005). Two noncentrosymmetric complexes: [W(CO)_4_(bipy)] and [W(CO)_4_(phen)] (bipy=2,2′-bipyridine, phen=1,10-phenanthroline) obtained through solvothermal synthesis and their optical properties. J. Organomet. Chem..

[102] Chapman J., Kolawole G., Long N., White A.J.P., Williams D.J., O′Brien P. (2005). Syntheses and X-ray structural analyses of the mononuclear tungsten hexacarbonyl complexes of 2,2′-bipyridine and 2,2′-bipyrimidine. S. Afr. J. Sci..

[103] Morgan G.T., Davies G.R. (1938). Residual affinity and co-ordination. XL. Some complex compounds of rhenium. J. Chem. Soc..

[104] Akimov V.M., Romero de Endara M., Babeshkina G.K., Stepanovich V.M., Dolganev V.P. (1971). Mono- and bis(α,α’-dipyridylium) hexachlororhenates. Izv. Vyssh. Uchebn. Zaved., Khim. Khim. Tek..

[105] Englert U., Koelle U., Nageswara R.N. (1994). Crystal structure of bis(2,2′-bipyridinium-(1-H))-hexachlororhenate, (C_10_H_9_N_2_)_2_ReCl_6_. Z. Krist. Cryst. Mater..

[106] Ferrari C. (1937). Analytical applications of α,α’-bipyridine. Ann. Chim. Appl..

[107] Ferrari C. (1937). Researches on the ferrotribipyridylic complex. Gazz. Chim. Ital..

[108] Barbieri G.A., Ferrari C. (1936). A reversible reaction between complex metalammine ions and hydrogen ions. Ric. Sci..

[109] Jaeger F.M., van Dijk J.A. (1934). Complex salts α,α’-bipyridyl with bivalent iron. Proc. K. Ned. Akad. Wet..

[110] Cambi L., Cagnasso A. (1933). The structure and magnetic susceptibility of complex ferrous compounds. Gazz. Chim. Ital..

[111] Simon A., Knauer H. (1939). Active iron. XII. Magnetic character of a few complex iron salts. Z. Elektrochem..

[112] Klemm W. (1931). Über physikalische Methoden im chemischen Laboratorium. XVIII. Die Bedeutung magnetischer Messungen für chemische Fragen. Angew. Chem..

[113] Klemm W. (1935). Die Bedeutung magnetischer Messungen für chemische Fragen. II. Angew. Chem..

[114] Cambi L., Cagnasso A. (1934). New types of complex paramagnetic salts of the iron series. Rend. Lombardo Sci..

[115] Barbieri G.A. (1934). Compounds intermediate between ferrocyanides and ferroamines. ATTI Accad. Naz. Lincei Cl. Sci. Fis. Mat. Nat. Rend..

[116] Jaeger F.M., van Dijk J.A. (1936). Die verschiedenen Typen von Komplexsalzen des α-α′-Dipyridyls mit Kupfer, Zink, Cadmium, Eisen, Nickel, Kobalt und Rhodium. Z. Anorg. Allg. Chem..

[117] Belamri Y., Setifi F., Francuski B.M., Novaković S., Zouaoui S. (2014). Crystal structure of tetraaqua(5,5′-dimethyl-2,2′-bipyridyl-κ2N,N′)iron(II) sulfate. Acta Crystallogr. Sect. E Struct. Rep. Online.

[118] Kuhn R., Wassermann A. (1933). Komplexbildung und Katalyse, hochaktive Zwischenstufen. Justus Liebigs Ann. Chem..

[119] Simon A., Haufe W. (1936). Über aktives Eisen. IX. Mitteilung. Über die αα′-Dipyridyl- und die o-Phenantrolinreaktion. Z. Anorg. Allg. Chem..

[120] Simon A., Reetz T. (1937). Über aktives Eisen XI. Mitteilung Die Ferrisalzkatalyse im System Oxalsäure-Hydroperoxyd und Oxalsäure-Sublimat-Hydroperoxyd. Z. Anorg. Allg. Chem..

[121] Manchot W., Pflaum W. (1933). Über den Mechanismus der Oxydationsvorgänge und die Oxydation von Eisen(II)salzen durch Wasserstoffsuperoxyd. Z. Anorg. Allg. Chem..

[122] Simon A., Morgenstern G., Arbrecht W.H. (1937). Über aktives Eisen X. Mitteilung. Über die magnetische Charakterisierung des Ferrimonodipyridylkomplexes und den Magnetismus einiger komplexer Ferropentacyanide. Z. Anorg. Allg. Chem..

[123] Shahbazi-Raz F., Notash B., Amani V., Safari N. (2016). 4,4′-Dimethyl-2,2′-bithiazole: Potent co-former in coordination compounds. Polyhedron.

[124] Eckenhoff W.T., Biernesser A.B., Pintauer T. (2012). Structural characterization and investigation of iron(III) complexes with nitrogen and phosphorus based ligands in atom transfer radical addition (ATRA). Inorg. Chim. Acta.

[125] Driessen W.L., de Graaff R.A.G., Vos J.G. (1983). Structure of fac-(2,2′-bipyridyl)trichloro(1H-1,2,4-triazole-N4)iron(III), [Fe(C_2_H_3_N_3_)(C_10_H_8_N_2_)Cl_3_]. Acta Crystallogr. Sect. C Cryst. Struct. Commun..

[126] Amani V., Safari N., Khavasi H.R., Mirzaei P. (2007). Iron(III) mixed-ligand complexes: Synthesis, characterization and crystal structure determination of iron(III) hetero-ligand complexes containing 1,10-phenanthroline, 2,2′-bipyridine, chloride and dimethyl sulfoxide, [Fe(phen)Cl_3_(DMSO)] and [Fe(bipy)Cl_3_(DMSO)]. Polyhedron.

[127] Figgis B.N., Reynolds P.A., Lehner N. (1983). *cis*-Bis(bipyridyl)dichloroiron(III) tetrachloroferrate(III), [Fe(bpy)_2_Cl_2_][FeCl_4_]; structure at 4.2 and at 115 K by neutron diffraction. Acta Crystallogr. Sect. B Struct. Sci..

[128] Figgis B.N., Patrick J.M., Reynolds P.A., Skelton B.W., White A.H., Healy P.C. (1983). Structural studies in the iron(III)/chloride/α,α’-diimine system. II. Ionic derivatives of stoichiometry FeCl_3_(phen,bpy)_1,1.5_. Aust. J. Chem..

[129] Figgis B.N., Reynolds P.A., White A.H. (1985). Covalent bonding in *cis*-[Fe(bpy)_2_Cl_2_][FeCl_4_] studied by x-ray diffraction at 120 K. Inorg. Chem..

[130] Witten E.H., Reiff W.M., Lazar K., Sullivan B.W., Foxman B.M. (1985). The ferric chloride-.alpha.-diamine system. 3. X-ray crystallographic, magnetic susceptibility, and zero- and high-field Moessbauer spectroscopy investigation of bis(2,2′-bipyridine)dichloroiron(2+) tetrachloroferrate(2–): Slow paramagnetic relaxation and magnetic ordering of complex bimetallic salts. Inorg. Chem..

[131] Morgan G.T. (1935). Recent researches on certain of the rarer elements. J. Chem. Soc..

[132] Burstall F.H. (1936). Optical activity dependent on co-ordinated bivalent ruthenium. J. Chem. Soc..

[133] Morgan G.T., Burstall F.H. (1938). Residual affinity and co-ordination. XXXIX. Complex ruthenium derivatives containing nitric oxide and polypyridyls. J. Chem. Soc..

[134] Haukka M., Venalainen T., Ahlgren M., Pakkanen T.A. (1995). Reactions of [Ru(bpy)(CO)_2_Cl_2_] in Acidic Media: Formation and Structural Characterization of [Ru(bpy)Cl_3_(NO)], [Ru_2_N(bpy)_2_Cl_5_(H_2_O)], and (H_5_O_2_)[Ru_2_N(bpy)_2_Cl_6_]. Inorg. Chem..

[135] Nagao H., Ooyama D., Howell F.S., Mukaida M., Mizumachi K. (1998). Crystal Structure of Bis-(2,2′- bipyridine)chloronitrosylruthenium(II) Perchlorate. Anal. Sci..

[136] Nagao H., Nishimura H., Funato H., Ichikawa Y., Howell F.S., Mukaida M., Kakihana H. (1989). Synthesis, properties, and molecular structure of trans-chloro(nitrosyl)bis(2,2′-bipyridine)ruthenium(2+): Trans and cis isomer characteristics compared. Inorg. Chem..

[137] Godward L.N.W., Wardlaw W. (1938). The Valency and Covalency of Ruthenium in the Blue Chloride Solution. J. Chem. Soc..

[138] Durham B., Cox D.I., Cordes A.W., Barsoum S. (1990). Structure of potassium (2,2′-bipyridine)tetrachlororuthenate(III). Acta Crystallogr. Sect. C Cryst. Struct. Commun..

[139] Eskelinen E., Da Costa P., Haukka M. (2005). The synthesis and electrochemical behavior of ruthenium(III) bipyridine complexes: [Ru(dcbpy)Cl_4_]^−^ (dcbpy=4,4′-dicarboxylic acid-2,2′-bipyridine) and [Ru(bpy)Cl_3_L] (L=CH_3_OH, PPh_3_, 4,4′-bpy, CH_3_CN). J. Electroanal. Chem..

[140] Seddon K.R., Seddon E.A. (2008). The Chemistry of Ruthenium.

[141] Jaeger F.M., van Dijk J.A. (1936). Complex salts of bipyridyl with bivalent and trivalent cobalt. Proc. K. Ned. Akad. Wet..

[142] Arun Kumar K., Amuthaselvi M., Dayalan A. (2011). cis-Bis(2,2′-bipyridine-κN,N’)dichloridocobalt(II) trihydrate. Acta Crystallogr. Sect. E Struct. Rep. Online.

[143] Ciornea V., Mingalieva L., Costes J.-P., Novitchi G., Filippova I., Galeev R.T., Shova S., Voronkova V.K., Gulea A. (2008). Structural determinations, magnetic and EPR studies of complexes involving the Cr(OH)2Cr unit. Inorg. Chim. Acta.

[144] Andersen P., Josephsen J., Lindberg B., Svensson S., Koskikallio J., Kachi S. (1971). Configurational Correlations of cis-Bis(2,2′-bipyridine) and of cis-Bis(1,10-phenanthroline) Complexes of Trivalent Metals by Means of X-Ray Powder Photographs. Acta Chem. Scand..

[145] Strenger I., Rosu T., Negoiu M. (2000). Refinement of the crystal structure of cw-bis(2,2′-bipyridyl)- dichlorocobalt(III) chloride dihydrate, [C_20_H1_6_N_4_CoC_l2_]Cl·2H_2_O. Z. Kristallogr. New Cryst. Struct..

[146] Niederhoffer E.C., Martell A.E., Rudolf P., Clearfield A. (1982). Structures of (carbonato)bis(2,2′-bipyridine)cobalt(III) and (carbonato)bis(1,10-phenanthroline)cobalt(III) complexes. Inorg. Chem..

[147] Li X.-X., Cao Z.-S., Jiang W.-J., Cai T.-J. (2007). Synthesis and determination of structure of [Co(2)CO_3_]NO_3_·5H_2_O. Hunan Keji Daxue Xuebao Ziran Kexueban.

[148] Jaeger F.M., van Dijk J.A. (1934). Complex salts of trivalent rhodium with 2,2′-bipyridyl. Proc. K. Ned. Akad. Wet..

[149] Lahuerta P., Latorre J., Martinez-Manez R., Garcia-Granda S., Gomez-Beltran F. (1991). Structure of bis(2,2′-bipyridine)dichlororhodium(III) chloride dihydrate. Acta Crystallogr. Sect. C Cryst. Struct. Commun..

[150] Morgan G.T., Burstall F.H. (1931). Optical activity dependent on co-ordinated nickel. Nature.

[151] Morgan G.T., Burstall F.H. (1931). Residual affinity and co-ordination. Part XXXIII. Optical activity dependent on coordinated nickel. J. Chem. Soc..

[152] Pfeiffer P., Tappermann F. (1933). Dipyridyl- und phenanthrolinhaltige Komplexsalze zweiwertiger Metalle. Z. Anorg. Allg. Chem..

[153] Xia L., Zhu Y., Wang Y.-M., Liu P., Xie J.-M. (2016). Syntheses, Structures, Fluorescence and Heterogeneous Catalysis of Coordination Polymers with 4-Benzoimidazol-1-yl-methyl Benzoic Acid and 2,2′-Dipyridine. Jiegou Huaxue.

[154] Boonlue S., Theppitak C., Chainok K. (2012). Tetra-aqua-(2,2′-bipyridine-κ^2^N,N’)nickel(II) sulfate. Acta Crystallogr. Sect. E Struct. Rep. Online.

[155] Pfeiffer P., Nakatsuka Y. (1933). Aktivierung von Komplexsalzen in wäßriger Lösung (II. Mitteil.). Ber. Dtsch. Chem. Ges. B.

[156] Jaeger F.M., van Dijk J.A. (1935). On some complex dipyridyl-salts of nickel and copper. Proc. K. Akad. Wet. Amst..

[157] Pedireddi V.R., Shimpi M.R., Yakhmi J.V. (2006). Room-Temperature Ionic Liquids: For a Difference in the Supramolecular Synthesis. Macromol. Symp..

[158] Charron G., Malkin E., Rogez G., Batchelor L.J., Mazerat S., Guillot R., Guihéry N., Barra A.L., Mallah T., Bolvin H. (2016). Unraveling σ and π Effects on Magnetic Anisotropy in cis-NiA_4_B_2_ Complexes: Magnetization, HF-HFEPR Studies, First-Principles Calculations, and Orbital Modeling. Chem. Eur. J..

[159] Garai M., Dey D., Yadav H.R., Choudhury A.R., Kole N., Biswas B. (2017). Catalytic aspects of a nickel(II)–bipyridine complex towards phosphatase and catechol dioxygenase activity. Polyhedron.

[160] Ikotun O.F., Ouellette W., Lloret F., Julve M., Doyle R.P. (2007). Synthesis, X-ray Structure, Thermal and Magnetic Behavior of [(bipy)_2_Ni_2_(μ-Cl)_2_Cl_2_(H_2_O)_2_]: The First Neutral Ferromagnetically Coupled Six-Coordinate Dichlorido-Bridged Nickel(II) Dimer. Eur. J. Inorg. Chem..

[161] Vasileiadou E., Angaridis P.A., Raptis R.G., Mathivathanan L. (2016). Aquabis(2,2′-bipyridine-κ2N,N′)chloridonickel(II) chloride chloroform monosolvate hemihydrate. IUCrData.

[162] Boutebdja M., Beghidja A., Beghidja C., Setifi Z., Merazig H. (2014). Bis(2,2′-bipyridyl-κ(2) N,N’)chloridonickel(II) nitrate trihydrate. Acta Crystallogr. Sect. E Struct. Rep. Online.

[163] Cambi L., Cagnasso A. (1934). Complexes of metals of the first transition series with dipyridyl and phenanthroline. ATTI Accad. Naz. Lincei Cl. Sci. Fis. Mat. Nat. Rend..

[164] Mann F.G., Purdie D. (1936). The constitution of complex metallic salts. IV. The constitution of certain bridged dipalladium derivatives. A novel type of tautomerism. J. Chem. Soc..

[165] Chatt J., Mann F.G. (1938). The constitution of complex metallic salts. Part VIII. The bridged thio-derivatives of palladous halides with tertiary phosphines. J. Chem. Soc..

[166] Maekawa M., Munakata M., Kitagawa S., Nakamura M. (1991). Crystal Structure of (2,2′-Bipyridine)dichloropalladium(II). Anal. Sci..

[167] Canty A.J., Skelton B.W., Traill P.R., White A.H. (1992). Structural Chemistry of the Platinum Group-Metals: MCl_2_(bpy) (M = Pd, Pt, bpy = 2,2′-Bipyridine). Aust. J. Chem..

[168] Kim N.-H., Hwang I.-C., Ha K. (2009). Redetermination of (2,2′-bipyridine-κ2N,N′)dichloridopalladium(II) dichloromethane solvate. Acta Crystallogr. Sect. E Struct. Rep. Online.

[169] Gutiérrez Márquez R.A., Crisóstomo-Lucas C., Morales-Morales D., Hernández-Ortega S. (2014). (2,2′-Bi-pyridine-κ(2) N,N’)di-chloridopalladium(II) 1,4-dioxane hemisolvate. Acta Crystallogr. Sect. E Struct. Rep. Online.

[170] Rosenblatt F., Schleede A. (1933). Der räumliche Bau der Platin-tetrammin-salze. Justus Liebigs Ann. Chem..

[171] Hazell A.C. (1984). Bis 2,2′-bipyridine platinum(II) dinitrate monohydrate. Acta Crystallogr. Sect. A Found. Crystallogr..

[172] Hazell A., Simonsen O., Wernberg O. (1986). Complexes of 2,2′-bipyridine (bpy) and 1,10-phenanthroline (phen) with platinum(II). Structures of [Pt^II^(bpy)_1.3_(phen)_0.7_](NO3)_2.0.3_H_2_O and [PtII(bpy)_2_](NO_3_)2.H_2_OActa Crystallogr. Sect. C Cryst. Struct. Commun..

[173] Palmans R., MacQueen D.B., Pierpont C.G., Frank A.J. (1996). Synthesis and Characterization of Bis(2,2′-bipyridyl)platinum(I): A Novel Microtubular Linear-Chain Complex. J. Am. Chem. Soc..

[174] Fedotova T.N., Minacheva L.K., Kuznetsova G.N. (2003). Formation of new platinum blues by the reaction of platinum(III) acetamidate chloride [Pt_2_(μ-NHCOCH_3_)_4_Cl_2_] with 2,2′-bipyridine: The crystal structure of [Pt(Bipy)_2_](CF_3_SO_3_)_2_. Zh. Neorg. Khim..

[175] Clare B.R., McInnes C.S., Blackman A.G. (2005). Bis(2,2′-bi-pyridine-κ2N,N′)platinum(II) bis--(perchlorate). Acta Crystallogr. Sect. E Struct. Rep. Online.

[176] Hudson T.A., Robson R. (2009). A New Class of TCNQ Derivatives Easily Generated from TCNQH_2_ Containing Discrete TCNQ2^−^Anions and Noncoordinating Cations. Cryst. Growth Des..

[177] Stork J.R., Rios D., Pham D., Bicocca V., Olmstead M.M., Balch A.L. (2005). Metal−Metal Interactions in Platinum(II)/Gold(I) or Platinum(II)/Silver(I) Salts Containing Planar Cations and Linear Anions. Inorg. Chem..

[178] Endres H., Keller H.J., Moroni W., Nöthe D., Dong V. (1978). The 1:3 radical salt bis(2,2′-dipyridyl)platinum(II)–7,7,8,8-tetracyanoquinodimethane, [Pt(dipy)_2_]^2+^[TCNQ]_3_^2−^. Acta Crystallogr. Sect. B Struct. Crystallogr. Cryst. Chem..

[179] Dong V., Endres H., Keller H.J., Moroni W., Nöthe D. (1977). Dimerization of 7,7,8,8-tetracyanoquinodimethane (TCNQ) radical anions via σ-bond formation: Crystal structure and EPR properties of bis(dipyridyl)platinum(II)–TCNQ, [Pt(2,2′-dipy)_2_
^2+^(TCNQ)_2_
^2−^]. Acta Crystallogr. Sect. B Struct. Crystallogr. Cryst. Chem..

[180] Chieh P.C. (1972). Crystal and molecular structure of aquobis-(2,2′-bipyridyl)palladium dinitrate. J. Chem. Soc. Dalton Trans..

[181] Hinamoto M., Ooi S., Kuroya H. (1972). Crystal structure of bis-(2,2′-bipyridyl)palladium(II) nitrate monohydrate and the stereochemistry of palladium(II). J. Chem. Soc. Chem. Commun..

[182] Gao E.J., Zhang Y.X., Zhu M.C., Liu H.Y., Huang Y., Zhang M., Su M., Guo M.J., Guan F., Gao X.N. (2010). Synthesis and characterization of a novel complex [Pd(Bipy)_2_](Bpcc)·11H_2_O. Russ. J. Coord. Chem..

[183] Henling L.M., Marsh R.E. (2014). Some more space-group corrections. Acta Crystallogr. Sect. C Cryst. Struct. Commun..

[184] Maeda S., Nishida Y., Okawa H., Kida S. (1986). The Crystal Structure of Bis(2,2′-bipyridyl)palladium(II) Picrate [Pd(bpy)2](pic)2. A Novel “Bow” Distortion. Bull. Chem. Soc. Jpn..

[185] Geremia S., Randaccio L., Mestroni G., Milani B. (1992). Bow–step and twist conformations and stacking interactions in palladium bipyridine and phenanthroline complexes. J. Chem. Soc. Dalton Trans..

[186] Stoccoro S., Alesso G., Cinellu M.A., Minghetti G., Zucca A., Bastero A., Claver C., Manassero M. (2002). New complexes of palladium(II) with chelating heterocyclic nitrogen ligands. J. Organomet. Chem..

[187] Milani B., Anzilutti A., Vicentini L., Sessanta o Santi A., Zangrando E., Geremia S., Mestroni G. (1997). Bis-Chelated Palladium(II) Complexes with Nitrogen-Donor Chelating Ligands Are Efficient Catalyst Precursors for the CO/Styrene Copolymerization Reaction. Organometallics.

[188] Wehman P., Dol G.C., Moorman E.R., Kamer P.C.J., van Leeuwen P.W.N.M., Fraanje J., Goubitz K. (1994). Ligand Effects in the Palladium-Catalyzed Reductive Carbonylation of Nitrobenzene. Organometallics.

[189] Yue C.-Y., Jiang F.-L., Yuan D.-Q., Chen L., Wu M.-Y., Hong M.-C. (2008). Synthesis, Crystal Structure and Photoluminescent Property of a 3D Hydrogen-bonded Supramolecular Compound with Large Channels. Chin. J. Struct. Chem..

[190] Morgan G.T., Burstall F.H. (1934). Residual affinity and co-ordination. XXXIV. 2,2′-Bipyridyl platinum salts. J. Chem. Soc..

[191] Randall J.T. (1938). Luminescence of solids at low temperatures. Nature.

[192] Osborn R.S., Rogers D. (1974). Crystal structure of the red form of 2,2′-bipyridyldichloroplatinum(II). J. Chem. Soc. Dalton Trans..

[193] Connick W.B., Henling L.M., Marsh R.E., Gray H.B. (1996). Emission Spectroscopic Properties of the Red Form of Dichloro(2,2′-bipyridine)platinum(II). Role of Intermolecular Stacking Interactions. Inorg. Chem..

[194] Falvello L.R., Garde R., Miqueleiz E.M., Tomás M., Urriolabeitia E.P. (1997). Evidence of C-H activation of acetone by a platinum(II) complex. Synthesis and structural characterization of [Pt(CH_2_COCH_3_)Cl(bipy)] (bipy = 2,2′-bipyridyl). Inorg. Chim. Acta.

[195] Textor M., Oswald H.R. (1974). Röntgenographische und spektroskopische Untersuchungen an zwei Modifikationen von Dichloro-2,2′-dipyridyl-platin(II). Zeit. Anorg. Allg. Chem..

[196] Canty A.J., Gardiner M.G., Jones R.C., Sharma M. (2011). Structural Chemistry of [MX2(bipy)] (M = Pd, Pt; X = Cl, Br, I): The Yellow Polymorph of Dichlorido(2,2′-bipyridine)platinum(II) and Diiodido(2,2′-bipyridine)palladium(II), and Overview of this System. Aust. J. Chem..

[197] Kato M., Kosuge C., Yano S., Kimura M. (1997). π-Stacking of [Pt(2,2′-bipyridine)(ethylenediamine)]^2+^as its Hexafluorophosphate Salt. Acta Crystallogr. Sect. C Cryst. Struct. Commun..

[198] Kato M., Takahashi J., Sugimoto Y., Kosuge C., Kishi S., Yano S. (2001). Selective formation of integrated stacks of (α-diimine)(ethylenediamine)platinum(II) and neutral π systems of the phenanthrene type. J. Chem. Soc. Dalton Trans..

[199] Masuda H., Yamauchi O. (1987). A structural basis for nucleic base‚Äîmetallointercalator lnteractions: Crystal structure of [Pt(2,2′-bipyridine)(ethylenediamine)].(AMP).10H_2_O (AMP = adenosine 5′-monophosphate). Inorg. Chim. Acta.

[200] Cavigliasso G., Stranger R., Lo W.K.C., Crowley J.D., Blackman A.G. (2013). The nature of species derived from [Pt(bipy)_2_]^2+^ in aqueous solution: X-ray structural, mass spectral, NMR, and computational studies. Polyhedron.

[201] Rotondo E., Bruschetta G., Bruno G., Rotondo A., Di Pietro M.L., Cusumano M. (2003). NMR Investigation and Dynamic Behaviour of [2,2′-Bipyridylbis(pyridine)platinum(II)]^2+^ and Related Cationic Complexes—Crystal Structure of [Pt(bipy)(py)_2_](PF_6_)_2_. Eur. J. Inorg. Chem..

[202] Corbo R., Georgiou D.C., Wilson D.J., Dutton J.L. (2014). Reactions of [PhI(pyridine)_2_]^2+^ with model Pd and Pt II/IV redox couples. Inorg. Chem..

[203] Reihlen H., Seipel G., Weinbrenner E. (1935). Über das asymmetrische Platinatom. VII. Eine neue Art optisch aktiver Verbindungen. Justus Liebigs Ann. Chem..

[204] Reihlen H., Hühn W. (1935). Über das asymmetrische Platinatom. VI. Justus Liebigs Ann. Chem..

[205] Tartarini G. (1933). New color reactions of cuprous salts. Gazz. Chim. Ital..

[206] Mann F.G., Purdie D., Wells A.F. (1936). The constitution of complex metallic salts. Part V. The constitution of the phosphine and arsine derivatives of cuprous iodide. The configuration of the co-ordinated cuprous complex. J. Chem. Soc..

[207] Skelton B.W., Waters A.F., White A.H. (1991). Lewis-Base Adducts of Group 11 Metal(I) Compounds. LXIII. Stereochemistries and Structures of the 1:1 Adducts of Cu^1^X (X = Cl, Br, I) With 2,2′-Bipyridine. Aust. J. Chem..

[208] Huang P.C., Parthasarathy K., Cheng C.H. (2013). Copper-catalyzed intramolecular oxidative C-H functionalization and C-N formation of 2-aminobenzophenones: Unusual pseudo-1,2-shift of the substituent on the aryl ring. Chem. Eur. J..

[209] Jaeger F.M., van Dijk J.A. (1934). Complex salts of α,α’-dipyridyl with bivalent copper. Proc. K. Ned. Akad. Wet..

[210] Tedenac J.C., Phung N.D., Avinens C., Maurin M. (1976). Stereochimie du motif de coordination dans les complexes a ligands mixtes du type M^II^(bipy)(H_2_O)_2_AB_4_ (M^II^ = Ni^II^ ou Cu^II.^; AB_4_^2-^ = SO_4_^2–^ ou BeF_4_^2–^) bipy: 2,2′-bipyridine. J. Inorg. Nucl. Chem..

[211] Chattopadhyay S.K., Mak T.C.W. (2000). Study of Cu^2+^ mediated oxidation of thiosemicarbazide, thiocarbohydrazide and thiourea. Inorg. Chem. Commun..

[212] Zheng Y.-Q., Lin J.-L. (2003). Crystal Structures of [Cu_2_(bpy)_2_(H_2_O)(OH)_2_(SO_4_)].4H_2_O and [Cu(bpy)(H_2_O)_2_]SO_4_ with bpy = 2,2′-Bipyridine. Z. Anorg. Allg. Chem..

[213] Chattopadhyay S.K., Seth S., Mak T.C.W. (2002). Studies of Copper(II) Thiosemicarbazide Complexes and their Reactivities. X-Ray Structure of Two Unusual Reaction Products, [Cu(bpy)(H_2_O)_2_SO_4_] and [(bpy)_2_Cu_2_(C_2_O_4_)Cl_2_].H_2_O. J. Coord. Chem..

[214] Clemente D.A., Marzotto A. (2004). 30 Space-group corrections: Two examples of false polymorphism and one of incorrect interpretation of the fine details of an IR spectrum. Acta Crystallogr. Sect. B Struct. Sci..

[215] Arumugam M., Kuppukannu R., Gabriele B., Andrea C. (2010). Synthesis, spectral and single crystal X-ray structural studies on bis(2,2′-bipyridine)sulphido M(II) (M=Cu, Zn) and diaquo 2,2′-bipyridine zinc(II)sulphate dihydrate. J. Serb. Chem. Soc..

[216] Tedenac J.-C., Philippot E. (1975). Structure du sulfate de bisaquomonobipyridyl-cuivre Cu(C_10_H_8_N_2_)H_2_O)_2_SO_4_. J. Inorg. Nucl. Chem..

[217] Hernández-Molina M., González-Platas J., Ruiz-Pérez C., Lloret F., Julve M. (1999). Crystal structure and magnetic properties of the single-μ-chloro copper(II) chain [Cu(bipy)Cl_2_] (bipy = 2,2′-bipyridine. Inorg. Chim. Acta.

[218] Garland M.T., Grandjean D., Spodine E., Atria A.M., Manzur J. (1988). Structures of catena-di-[mu]-chloro- and catena-di-[mu]-bromo-(2,2-bipyridine)copper(II). Acta Crystallogr. Sect. C Cryst. Struct. Commun..

[219] Wang Y.-Q., Bi W.-H., Li X., Cao R. (2004). (2,2′-Bi-pyridine-κ2N,N′)-di-chloro-copper(II). Acta Crystallogr. Sect. E Struct. Rep. Online.

[220] Garai M., Dey D., Yadav H.R., Choudhury A.R., Maji M., Biswas B. (2017). Catalytic Fate of Two Copper Complexes towards Phenoxazinone Synthase and Catechol Dioxygenase Activity. Chem. Sel..

[221] Liu E., Zhang Y., Li L., Yang C., Fettinger J.C., Zhang G. (2015). New copper(II) species from the copper/2,2′-bypyridine and copper/4-dimethylaminopyridine catalyzed aerobic alcohol oxidations. Polyhedron.

[222] Kostakis G.E., Nordlander E., Haukka M., Plakatouras J.C. (2006). Di-μ-chloro-bis-[(2,2-bi-pyridine)chloro-copper(II)]. Acta Crystallogr. Sect. E Struct. Rep. Online.

[223] Jaeger F.M., van Dijk J.A. (1934). Complex salts of α,α’-dipyridyl with bivalent copper. II. Proc. K. Ned. Akad. Wet..

[224] Kumar D., Kapoor I.P.S., Singh G., Fröhlich R. (2012). Preparation, characterization, and kinetics of thermolysis of nickel and copper nitrate complexes with 2,2′-bipyridine ligand. Thermochim. Acta.

[225] Nakai H., Ooi S., Kuroya H. (1977). The Crystal Structure of Nitratotriaquo(2,2′-bipyridine)copper(II) Nitrate [Cu(NO_3_)(H_2_O)_3_(bipy)]NO_3_. Bull. Chem. Soc. Jpn..

[226] Mathews I.I., Manohar H. (1991). Redetermination of the structure of triaqua(2,2′-bipyridine)nitratocopper(II) nitrate. Acta Crystallogr. Sect. C Cryst. Struct. Commun..

[227] Jianmin L., Jianbin Z., Yanxiong K., Xintao W. (1996). A Novel Structure of Bipyridine Coordinated with Copper(II): [Cu(bipy)(H_2_O)_3_]·(NO_3_)_2_. Cryst. Res. Technol..

[228] Potapov A.S., Domina G.A., Petrenko T.V., Khlebnikov A.I. (2012). Synthesis and crystal structure of discrete complexes and coordination polymers containing 1,3-bis(pyrazol-1-yl)propane ligands. Polyhedron.

[229] Marjani K., Davies S.C., Durrant M.C., Hughes D.L., Khodamorad N., Samodi A. (2005). Bis(2,2′-bi-pyridine)-nitratocopper(II) nitrate. Acta Crystallogr. Sect. E Struct. Rep. Online.

[230] Lobana T.S., Rani A., Jassal A.K., Jasinski J.P. (2015). Reactivity of thiazolidine-2-thione towards CuI/CuII: Synthesis and structures of [3-(2-thiazolin-2-yl)thiazolidine-2-thione]copper(I) bromide and [bis(2,2′-bipyridine)nitratocopper(II)] nitrate. J. Chem. Sci..

[231] Proctor I.M., Stephens F.S. (1969). An example of a cis-distorted octahedral copper(II) complex: The crystal structure of nitritobis-2,2′-bipyridylcopper(II) nitrate. J. Chem. Soc. A.

[232] Simmons C., Clearfield A., Fitzgerald W., Tyagi S., Hathaway B. (1983). The X-ray crystal structure and electronic properties of [Cu(bipy)_2_(ONO)][NO_3_](bipy = 2,2′-bipyridyl) at 298 and 165 K, a fluxional cis-distorted octahedral CuN_4_O_2_ chromophore. J. Chem. Soc. Chem. Commun..

[233] Simmons C.J., Hathaway B.J., Amornjarusiri K., Santarsiero B.D., Clearfield A. (1987). The first determination of the energy difference between solid-state conformers by x-ray diffraction. 1. The crystal structure of the pseudo-Jahn-Teller complex (nitrito)bis(2,2′-bipyridyl)copper(II) nitrate at 20, 100, 165 and 296 K and of its isostructural zinc(II) analog at 295 K. 2. The possibility of using x-ray diffraction to characterize adiabatic potential energy surfaces and relative ligand strengths. J. Am. Chem. Soc..

[234] Simmons C.J., Clearfield A., Fitzgerald W., Tyagi S., Hathaway B.J. (1983). Fluxional behavior of a pseudo-Jahn-Teller complex: X-ray crystal structure of [Cu(bppy)_2_(ONO)][NO_3_] at 165 and 296 K. Inorg. Chem..

[235] Fereday R.J., Hodgson P., Tyagi S., Hathaway B.J. (1981). Crystal structure and electronic properties of bis(2,2′-bipyridyl)-nitratocopper(II) nitrate monohydrate. J. Chem. Soc. Dalton Trans..

[236] Nakai N. (1980). The Crystal Structure of Bis(2,2′-bipyridine)nitratocopper(II) Nitrate Monohydrate [Cu(NO_3_)(bpy)_2_]NO_3_·H_2_O. Bull. Chem. Soc. Jpn..

[237] Catalan K.J., Jackson S., Zubkowski J.D., Perry D.L., Valente E.J., Feliu L.A., Polanco A. (1995). Copper(II) nitrate compounds with heterocyclic ligands: Structures of [Cu(NO_3_)(2,2′-dipyridyl)_2_][NO3]·H_2_O and [Cu(H_2_O)(1,10-phenanthroline)_2_][NO_3_]_2_. Polyhedron.

[238] Pfeiffer P., Christeleit W. (1937). Complex copper salts of alpha-amino acids. Stiasny Festschr..

[239] do Nascimento Neto J.A., da Silva C.C., Ribeiro L., Valdo A.K.S.M. (2018). Competition between coordination bonds and hydrogen bonding interactions in solvatomorphs of copper(II), cadmium(II) and cobalt(II) complexes with 2,2′-bipyridyl and acetate. Z. Kristallogr. Cryst. Mater..

[240] Perlepes S.P., Libby E., Streib W.E., Folting K., Christou G. (1992). The reactions of Cu_2_(O_2_CMe)_4_(H_2_O)_2_ with 2,2′-bipyridine (bpy): Influence of the Cu: Bpy ratio, and the structure of a linear polymer comprising two alternating types of Cu2 units. Polyhedron.

[241] Koo B.K. (2001). Synthesis, Crystal Structure, and Characterization of Copper(II) Acetate Complex. Bull. Kor. Chem. Soc..

[242] Morgan G.T., Burstall F.H. (1930). Researches on residual affinity and co-ordination. Part XXXII. Complex salts of bivalent silver. J. Chem. Soc..

[243] Sugden S. (1932). 20. Magnetism and valency. Part I. Copper and silver compounds. J. Chem. Soc..

[244] Barbieri G.A. (1932). The electrolytic preparation of several complex salts of bivalent silver. ATTI Accad. Naz. Lincei Cl. Sci. Fis. Mat. Nat. Rend..

[245] Kandaiah S., Huebner R., Jansen M. (2012). Electrocrystallisation and single crystal structure determination of Bis(2,2′-bipyridyl)silver(II) perchlorate [Ag(bipy)_2_](ClO_4_)_2_. Polyhedron.

[246] Atwood J.L., Simms M.L., Zatko D.A. (1973). Bis(2,2′-bipyridine)silver(II) Nitrate Monohydrate, Ag(N_2_C_10_H_8_)_2_.(NO_3_)_2_.H_2_O. Cryst. Struct. Commun..

[247] Morgan G.T., Sugden S. (1931). Paramagnetism of Bivalent Silver. Nature.

[248] McMillan J.A., Smaller B. (1961). Paramagnetic Resonance of Some Silver (II) Compounds. J. Chem. Phys..

[249] Cervone E. (1962). Electronic spectra of some Ag^++^ complexes. Annali Chimica.

[250] Thorpe W.G., Kochi J.K. (1971). Silver(II) complexes of dipyridyl. J. Inorg. Nucl. Chem..

[251] Hieber W., Schulten H. (1937). Über Metallcarbonyle. XXIV. Bildungsweise und Metallsalzreaktionen des Kobaltcarbonylwasserstoffs. Z. Anorg. Allg. Chem..

[252] Dothie H.J., Llewellyn F.J., Wardlaw W., Welch A.J.E. (1939). Stereochemistry of quadricovalent atoms: Gold. J. Chem. Soc..

[253] Jones P.G., Clegg A., Sheldrick G.M. (1980). Potassium Dicyanoaurate(I)-2,2′-Bipyridyl. Acta Crystallogr. Sect. B Struct. Crystallogr. Cryst. Chem..

[254] Döring C., Strey M., Jones P.G. (2017). The march of progress: Structures of the adducts of potassium dicyanidoaurate(I) with 2,2′-bipyridyl (redetermination) and 1,10-phenanthroline. Acta Crystallogr. Sect. C Struct. Chem..

[255] Brain F.H., Gibson C.S. (1939). Organic compounds of gold. VII. Methyl and ethyl compounds. J. Chem. Soc..

[256] Pfeiffer P., Quehl K. (1932). Aktivierung von Komplexsalzen in wäßriger Lösung (II. Mitteil.). Ber. Dtsch. Chem. Ges. B.

[257] Pfeiffer P., Quehl K. (1931). Über einen neuen Effekt in Lösungen optisch-aktiver Substanzen (I. Mitteil.). Ber. Dtsch. Chem. Ges. B.

[258] Jaeger F.M., van Dijk J.A. (1934). Complex salts of α,α’-bipyridyl with zinc and cadmium. Proc. K. Ned. Akad. Wet..

[259] Harvey M., Baggio S., Mombrú A., Baggio R. (2000). Three new Zn^II^ sulfate complexes. Acta Crystallogr. Sect. C Cryst. Struct. Commun..

[260] Behera J.N., Rao C.N. (2005). Organically-Templated Zinc and Thorium Sulfates with Chain and Layered Structures. Z. Anorg. Allg. Chem..

[261] Lan C.-L., Zhang S.-H., Zhou Z.-Y. (2007). Synthesis and crystal structure of one-dimensional coordination polymers [Zn(bipy)(H_2_O)_2_SO_4_]. Chem. Res. Appln..

[262] Karmakar A., Baruah J.B., Shankar R.B. (2009). Zinc(II) and cobalt(II) complexes of (3-carboxymethoxy-naphthalen-2-yloxy)-acetic acid: A structural study. CrystEngComm.

[263] Tai X.-S., Xu J., Feng Y.-M. (2009). Synthesis and crystal structure of 3D network Zn(II) complex. Huaxue Shiji.

[264] Wang L.H. (2011). Synthesis and Structural Characterization of Zn(II) Coordination Polymer Material. Adv. Mater. Res..

[265] Park H.-M., Hwang I.-H., Bae J.-M., Jo Y.-D., Kim C., Kim H.-Y., Kim Y.-M., Kim S.-J. (2012). Anion Effects on Crystal Structures of Cd^II^ Complexes Containing 2,2′-Bipyridine: Photoluminescence and Catalytic Reactivity. Bull. Korean Chem. Soc..

[266] Rodesiler P.F., Turner R.W., Charles N.G., Griffith E.A.H., Amma E.L. (1984). Solution and solid-state cadmium-113 NMR of Cd(alpha.alpha.’-bpy)_2_X_2_ (X = Cl^−^,Br^−^, NCS^−^,NO_3_^−^,H_2_O) and crystal structures of the nitrate (monohydrate) and the isothiocyanate derivatives. Inorg. Chem..

[267] Turner R.W., Rodesiler P.F., Amma E.L. (1982). The crystal structure, solid and solution ^113^Cd NMR of bis(α,α′-dipyridyl)cadmium(II) nitrate hemihydrate and its relationship to Cd containing proteins. Inorg. Chim. Acta.

[268] Jaeger F.M., van Dijk J.A. (1935). Complex salts of alpha-alpha’-bipyridyl with zinc and cadmium. II. Proc. Acad. Sci. Amst..

[269] Cherni S.N., Cherni A., Driss A. (2012). Crystal Structure of Bicapped Trigonal-Antiprismatic Coordinated Cd(II) Complex [Cd(C_10_H_8_N_2_)(NO_3_)_2_(H_2_O)]. X-Ray Struct. Anal. Online.

[270] Freire E., Baggio S., Baggio R., Suescun L. (1999). Mercury(II) halide complexes with N-donor organic ligands: Crystal and molecular structure of HgI_2_R, R = 1,10-phenanthroline, 2,9-dimethyl-1,10-phenanthroline, bipyridine. J. Chem. Crystallogr..

[271] Pfeiffer P., Christeleit W. (1938). Komplexsalze der Alkali- und Erdalkalimetalle. Z. Anorg. Allg. Chem..

[272] Snell F.D. (1921). Colorimetric Analysis.

[273] Rosenfeld L. (1999). Four Centuries of Clinical Chemistry.

[274] Hill R. (1930). Method for the estimation of iron in biological material. Proc. R. Soc. Lond. Ser. B.

[275] Feigl F., Hamburg H. (1931). The detection of iron. Z. Anal. Chem..

[276] Feigl F., Krumholz P., Hamburg H. (1932). Colorimetric determination of iron with α,α’-dipyridyl. Z. Anal. Chem..

[277] van Nieuwenburg C.J., Blumendal H.B. (1935). Cerimetric titration of small amounts of iron by means of α,α’-bipyridyl as indicator. Mikrochemie.

[278] Poluektov N.S., Nazarenko V.A. (1937). An application of a ferrous bipyridyl complex in micro-chemical analysis. Zh. Prikl. Khim. (S. Peterburg Russ. Fed.).

[279] McFarlane W.D. (1936). Determination of iron by titanium titration and by alpha,alpha-bipyridine colorimetry—Improved analytical procedure. Ind. Eng. Chem. Anal. Ed..

[280] Kohler G.O., Elvehjem C.A., Hart E.B. (1936). Modifications of the bipyridine method for available iron. J. Biol. Chem..

[281] Szebelledy L., Ajtai M. (1937). Detection of iron. Magy. Gyogyszeresztud. Tarsasag Ert..

[282] Thiel A., van Hengel E. (1937). Grundlagen und Anwendungen der Absolutcolorimetrie, XVI. Mitteil.: Über die absolutcolorimetrische Bestimmung des Eisens. Ber. Dtsch. Chem. Ges. B.

[283] Szelenyi T. (1938). Methods of quantitative spectral analysis. Magy. Mern. Epitesz-Egylet Kozl..

[284] Nießner M., Wien T.H. (1939). Mikroanalytische Forschungen in der Metallkunde. Angew. Chemie.

[285] Klockmann R. (1939). Newer organic reagents in medical-chemical analysis. E. Merck’s Jahresber..

[286] Hill R., Keilin D. (1933). Estimation of hematin iron and the oxidation-reduction equivalent of cytochrome c. Proc. R. Soc. Lond. Ser. B.

[287] Muller H. (1933). Die Verwendung von α-α′-Dipyridyl zur Bestimmung von Ferro- und Gesamteisen in Natürlichen Wässern. Mikrochemie.

[288] Cooper L.H.N. (1935). Iron in the sea and in marine plankton. Proc. R. Soc. Lond. Ser. B.

[289] Sirks H.A. (1939). The determination of the iron content of water used in butter making. Versl. Landbouwkd. Onderz. C.

[290] Hutchinson G.E., Deevey E.S., Wollack A. (1939). The oxidation-reduction potentials of lake waters and their ecological significance. Proc. Natl. Acad. Sci. USA.

[291] Pal J.C. (1939). Ionizable iron in cow and human milk. Indian Med. Gaz..

[292] Bode G. (1933). Estimation of iron in beer by means of α,α’-bipyridyl. Wochenschr. Brau..

[293] Gray P.P., Stone I.M. (1938). Direct determination of iron in malt beverages. Ind. Eng. Chem. Anal. Ed..

[294] Kok J.A.F. (1933). Removal of metals from foods. Acta Brevia Neerl. Physiol. Pharmacol. Microbiol..

[295] Elvehjem C.A., Hart E.B., Sherman W.C. (1933). The availability of iron from different sources for hemoglobin formation. J. Biol. Chem..

[296] Ranganathan S. (1938). The available iron in some common Indian foodstuffs, determined by the α,α’-bipyridine method. Indian J. Med. Res..

[297] Harris R.S., Mosher L.M., Bunker J.W.M. (1939). The nutritional availability of iron in molasses. Am. J. Dig. Dis. Nutr..

[298] Yang E.F., Dju M.Y. (1939). The total and available iron in vegetable foods. Zhongguo Shenglixue Zazhi.

[299] Goswami H., Basu U. (1938). “Available” iron in Indian foodstuffs. Indian J. Med. Res..

[300] Smith M.C., Otis L. (1936). The banana as a source of iron for hemoglobin formation. J. Home Econ..

[301] Shackleton L., McCance R.A. (1936). The ionizable iron in foods. Biochem. J..

[302] Schlegel H. (1936). A critical investigation on the possibility of the determination of traces of iron in silicic acid, with special consideration of the quantitative spectrum analysis. Beiheft Z. Ver. Deut. Chem..

[303] Hackl O. (1937). Micro tests for ferrous and ferric oxides in silicates. Mikrochemie.

[304] Dyer W.J., McFarlane W.D. (1938). A study of the iron in a podzol soil by means of an improved bipyridine method. Can. J. Res. Sect. B.

[305] Kazarinova-Oknina V.A. (1938). Colorimetric determination of ferrous oxide in phosphate rocks. Zavod. Lab..

[306] Ignatieff V. (1937). Method for determining ferrous iron in soil solutions and a study of the effect of light on the reduction of iron by citrate and 2,2′-dipyridyl. J. Soc. Chem. Ind. Lond..

[307] Engel L.L. (1934). Method for the determination of iron in dental enamel. J. Dent. Res..

[308] Murray M.M., Glock G.E., Lowater F. (1939). Chemical and spectrographic determination of iron in tooth material. Br. Dent. J..

[309] Shorland F.B., Wall E.M. (1936). A rapid method for the estimation of total iron in blood. Biochem. J..

[310] Coombs H.I. (1936). The hemoglobin and iron in the blood. I. The determination of the total iron. Biochem. J..

[311] Wall E.M., Shorland F.B. (1936). The nature, properties and estimation of iron in blood. J. N. Z. Inst. Chem..

[312] Ferrari C. (1937). Histochemical identification of organic iron. Ann. Chim. Appl..

[313] Leuthardt F. (1939). Medicinal chemistry. Mitt. Geb. Lebensmittelunters. Hyg..

[314] Heilmeyer L., Plotner H. (1936). Iron deficiency and its treatment. Klin. Wochenschr..

[315] Lintzel W. (1933). Microdetermination of iron in biological materials. Z. Gesamte Exp. Med..

[316] Sherman W.C., Elvehjem C.A., Hart E.B. (1934). Further studies on the availability of iron in biological material. J. Biol. Chem..

[317] Scharrer K. (1934). The colorimetric estimation of iron in plant materials by means of α,α’-bipyridyl. Z. Pflanzenernaehr. Dueng. Bodenkd..

[318] Jackson S.H. (1938). Determination of iron in biological material. Ind. Eng. Chem. Anal. Ed..

[319] Sherman W.C., Elvehjem C.A., Hart E.B. (1934). Factors influencing the utilization of the iron and copper of egg yolk for hemoglobin formation. J. Biol. Chem..

[320] Kuhn A. (1934). Investigations of homeopathic iron preparations. Pharm. Zentralhalle Dtschl..

[321] Hahn P.F., Whipple G.H. (1938). Iron metabolism in experimental anemia. “Availability of iron”. J. Exp. Med..

[322] Kuhn R., Sörensen N.A., Birkofer L. (1940). Über die Eisenproteide der Milz; der Bauplan des Ferritins. Dtsch. Chem. Ges. (A/B Ser.).

[323] Schulek E., Floderer I. (1939). Colorimetric determination of ferrous and ferric ions in the presence of aluminum, manganese, zinc, mercury, copper, phosphoric acid and organic substances, with special reference to drug preparations. Magy. Gyogyszeresztud. Tarsasag Ert..

[324] Okac A. (1939). Effect of cuprous salts on Blau’s test for iron with α,α’-dipyridyl. Collect. Czech. Chem. Commun..

[325] Parker W.E., Griffin F.P. (1939). Some observations on the determination of iron and copper in biological material by photoelectric colorimetry. Can. J. Res. Sect. B.

[326] Koenig E.W. (1939). Direct determination of alumina in certain silicates. Ind. Eng. Chem. Anal. Ed..

[327] McFarlane W.D. (1936). The reduction of iron by tissue extracts and by ascorbic acid with a note on the stabilization of ascorbic acid solutions. Biochem. J..

[328] Thiel A., Heinrich H., van Hengel E. (1938). Basis and application of absolute colorimetry. XVII. Further experiences with the absolute colorimetry of iron. Ber. Dtsch. Chem. Ges. B.

[329] Shapiro M.Y. (1938). Sensitive test for tantalum and columbium. Zh. Prikl. Khim. (S.-Peterburg, Russ. Fed.).

[330] Kuster W., Erfle E., Roll E.V., Schiller K. (1926). Complex ferro salts. Z. Physiol. Chem..

[331] Schulek E., Floderer I. (1939). A new method for the colorimetric determination of vitamin C. Angew. Chem..

[332] Emmerie A., Engel C. (1939). Chemical structure and properties of tocopherol (vitamin E). IV. Chemical tests for the tocopherols. Soc. Chem. Ind. Food Group.

[333] Emmerie A., Engel C. (1939). Colorimetric determination of tocopherol (vitamin E). III. Estimation of tocopherol in blood serum. Recl. Trav. Chim. Pays-Bas.

[334] Emmerie A., Engel C. (1939). Colorimetric determination of tocopherol (vitamin E). II. Adsorption experiments. Recl. Trav. Chim. Pays-Bas.

[335] Emmerie A., Engel C. (1938). Colorimetric determination of α-tocopherol (vitamin E). Recl. Trav. Chim. Pays-Bas.

[336] Emmerie A., Engel C. (1938). Colorimetric determination of dl-α-tocopherol (vitamin E). Nature.

[337] John W. (1939). Über das Vitamin E. Angew. Chem..

[338] Weil L. (1935). The activation of arginase. J. Biol. Chem..

[339] Lintzel W. (1933). Demonstration of the absorption of food iron in the form of ferrous ions. Biochem. Z..

[340] Rossi A. (1939). Activation of arginase. Boll. Soc. Ital. Biol. Sper..

[341] Beccari E. (1938). Pharmacological studies on the ferrous tri-α,α’-bipyridyl complex. I. General toxicity. Boll. Soc. Ital. Biol. Sper..

[342] Beccari E. (1938). Pharmacological studies on the ferrous tri-α,α’-bipyridyl complex. II. Distribution in the organism. Boll. Soc. Ital. Biol. Sper..

[343] Michaelis L., Stern K.G. (1931). Influence of heavy metals and metal complexes on the proteolytic processes. Biochem. Z..

[344] Stern K.G. (1932). The catalase of colorless blood cells. Z. Physiol. Chem..

[345] Maschmann E., Helmert E. (1933). Inhibition of cathepsin and activation of papain by α-thiocarboxylic acids. Z. Physiol. Chem..

[346] Eichholtz F. (1939). New conceptions of the pharmacology and therapeutic use of iron. Med. Klin. (Muenchen Ger.).

[347] Bonino G.B., Ansidei R.M. (1934). Investigations on the Raman effect in some organic substances. arxiv.

[348] Yamasaki K. (1937). Absorptions spektren von Metallkomplexsalzen des 2,2′-Dipyridyls. I. Bull. Chem. Soc. Jpn..

[349] Yamasaki K. (1938). Absorptions spektren von Metallkomplexsalzen des 2,2′-Dipyridyls. II. Bull. Chem. Soc. Jpn..

[350] Franke I.W. (1932). Autoxidation of unsaturated fatty acids. Justus Liebigs Ann. Chem..

